# Dietary attribution to burden of chronic disease in Australia: a systematic analysis of the Australian Institute of health and welfare 2024 national burden of disease dataset

**DOI:** 10.1016/j.eclinm.2025.103418

**Published:** 2025-08-11

**Authors:** Yuanxin Xu, Jing Sun

**Affiliations:** a The Fourth Affiliated Hospital, Harbin Medical University, Harbin, Postcode 150000, China; b Rural Health Research Institute, Charles Sturt University, NSW, 2800, Australia; c School of Health Science and Social Work, Griffith University, Gold Coast Campus, LD 4215, Australia; d Data Science Institute, University of Technology Sydney, Sydney, NSW, 2007, Australia

**Keywords:** Burden of disease, Epidemiology, Dietary risks, Non-communicable chronic disease

## Abstract

**Background:**

Non-communicable chronic diseases (NCDs), including cardiovascular diseases, cancer, chronic kidney disease (CKD), and type 2 diabetes mellitus (T2DM), pose a significant burden on Australia's healthcare system. Despite advancements in disease prevention and management, NCDs remain the leading cause of morbidity and mortality. This study aimed to assess trends in the burden of NCDs and the impact of dietary risks in Australia from 2003 to 2024 using data from the Australian Institute of Health and Welfare (AIHW).

**Methods:**

Data were from the AIHW 2024 burden of disease dataset, which provided estimates for mortality, years lived with disability (YLDs), years of life lost (YLLs), and disability-adjusted life years (DALYs). Since the 2024 death counts were missing, total mortality attributable to dietary risks was expressed as the percentage change from 2003 to 2018. Joinpoint Regression was performed to assess DALYs, YLLs, and YLDs of NCDs from 2003 to 2024, analyze the contribution of various dietary risk factors to the disease burden and conduct subgroup comparisons based on sex.

**Findings:**

From 2003 to 2018, mortality attributed to dietary risks declined by 15.29%. From 2003 to 2024, dietary risks attributable to DALYs decreased by 16.93%. Atrial fibrillation showed the most significant decline in both mortality (−10.0%) and DALYs (−7.81%), driven by a reduction in high-sodium diets. In contrast, inflammatory heart disease experienced the highest increases in DALYs, rising by 18.18% percentage change, which is associated with diet high in sodium. Breast cancer showed the most significant growth driven by diet high in red meat in dietary attributable DALYs, with a percentage change of 6.45%; and in deaths, with a percentage change of 6.67%. T2DM also illustrated a slight increase in dietary attributable DALYs driven by a diet high in red meat, with a percentage change of 2.36%, and in deaths driven by a diet high in processed meat, with a percentage change of 4.10%. In contrast, CKD decreased due to reduction of high sodium in dietary attributable DALYs, with a percentage change of 1.49%, and deaths, with a percentage change of 3.13%. Males showed an increase in risks related to high sodium consumption, with inflammatory heart disease DALYs rising by 15.38%, and a rise in oesophageal cancer deaths linked to low vegetable intake, with a percentage change of 14.49%. Females experienced an increase in T2DM DALYs attributable to high red meat consumption, with a percentage change of 10.26%, and a significant rise in coronary heart disease deaths associated with high sodium intake, with a percentage change of 18.97%.

**Interpretation:**

These findings emphasize the ongoing impact of dietary risks on NCDs burden in Australia and underscore the need for sex-specific and targeted dietary interventions to reduce preventable NCDs. Strengthening public health policies, dietary guidelines, and awareness campaigns is crucial for mitigating the impact of poor diet on long-term health outcomes.

**Funding:**

10.13039/501100005104AIHW is primarily funded by the 10.13039/100015539Australian Government, with additional funding from state and territory governments.

**Research in context**

**Evidence before this study**

Chronic diseases, especially cardiovascular disease, cancer, chronic kidney disease, and type 2 diabetes mellitus, remain leading causes of death and disability in Australia. While existing research has established a clear link between dietary risks and non-communicable diseases, much of the available data is either outdated or fragmented. The Australian Institute of Health and Welfare (AIHW) regularly publishes national disease burden reports; however, there remains a lack of systematic analysis on dietary risk trends, particularly when subgrouped by sex.

**Added value of this study**

This study contained the AIHW 2024 disease burden dataset to analyze national data from 2003 to 2024, providing the latest overview of the role of Australian dietary risks in chronic disease burden, which not only refines the components of disease burden, such as years of life lost, disability-adjusted life years, and years lived with disability, but also highlights the influence of specific dietary factors, including processed meat consumption, high sodium intake, and insufficient whole grain consumption, revealing trends in different diseases and sex groups.

**Implications of all the available evidence**

The research further emphasizes the role of dietary risk factors in Australia's chronic disease landscape, and poor diets remain a major driver of preventable diseases and deaths. The study suggests that public health policies require refinement and sex-specific targeting and provides evidence to support the formulation of targeted dietary intervention measures, the optimization of national dietary guidelines, and the enhancement of public health promotion strategies.

## Introduction

Over the past century, Australia has made significant progress in controlling infectious diseases,^1^ continuously enhancing disease screening technologies and improving diagnosis and treatment methods.^2^ Australia has significantly reduced mortality rates, with children born in 2023 expected to live more than 80 years.^3^ Despite increased longevity, the burden of chronic diseases remains high. It is estimated that Australians will spend more than one-tenth of their lives in a state of illness,^4^ highlighting the profound impact of non-communicable chronic diseases (NCDs) on quality of life.

NCDs, including cardiovascular diseases (CVDs), cancer, kidney disease, and type 2 diabetes mellitus (T2DM), have become a significant challenge in Australia's healthcare.^5^ The years lived with disability (YLDs) due to chronic conditions rose from 1.6 million in 2003 to 2.5 million in 2023, over the same period, the years of life lost (YLLs) due to chronic conditions rose from 1.7 million to 1.9 million.^4^ NCDs have become the highest-cost disease category within the healthcare system. In 2020–2021, they accounted for 29% of Australia's healthcare expenditure, placing a substantial financial burden on the national economy.^6^ The causes and risk factors of NCDs are currently not comprehensively assessed. In particular, an unhealthy diet has been widely recognized as a significant global risk factor for NCDs, particularly CVDs, T2DM, and certain types of cancer.^7^ A global dietary analysis study found that unhealthy diets—such as high sodium intake, low whole grain consumption, and insufficient fruit intake—contribute to over 11 million deaths annually, the majority of which are related to CVDs.^8^ For individuals with CVDs, T2DM, or stroke, increasing the intake of fruits, vegetables, whole grains, and fish can reduce the risk of dementia by 31%.^9^ According to the Australian Dietary Guidelines, it is recommended to increase the daily consumption of grains, legumes, lean meats and fish, milk and dairy products, and fruits.^10^ Currently, no specific disease burden analysis is attributed to dietary risk factors for the Australian population, and the trend of disease burden from the 2000s to the present has not been examined.

While global efforts such as the Global Burden of Disease (GBD) study have assessed dietary risk factors at the international level,^11^ national analyses using locally validated data remain essential to inform country-specific interventions. This study aimed to fill this research gap by utilizing the latest 2024 data from the Australian Institute of Health and Welfare (AIHW) on NCDs mortality and disability-adjusted life years (DALYs) associated with dietary risk factors. By conducting an in-depth analysis of dietary risk factors from 2003 to 2024, this research examined their role in the progression and outcomes of NCDs in Australia. Through a detailed analysis of dietary factors across sex and NCDs, the study identified which specific dietary habits have contributed most to different types of NCDs conditions across male and female groups over 21 years. In addition, annual percentage changes over time relating to both the impacts of risk factors and the disease burden over time were analyzed.

## Methods

### Data source

Data in this study came from the AIHW database 2024,^12^ which is a nationally representative, population-level health database maintained by the government. Unlike the GBD database, which uses a standardized model for cross-country estimates, the AIHW relies on local Australian data inputs and modeling assumptions. While the GBD is useful for international comparisons, the AIHW provides higher local accuracy, making it more suitable for national policy evaluation, sex trend analysis, and public health intervention planning.^13^ The AIHW provides comprehensive and up-to-date estimates of the disease burden in Australia, including attributable burden and risk factors, categorized by sex and age group. This research assessed the burden of NCDs and the impact of dietary risks on mortality and DALYs in Australia from 2003 to 2024. We used data on annual deaths, DALYs, YLLs, and YLDs related to NCDs from publicly available Australian health databases. We confirm that the dietary risk-attributed DALY, YLL and YLD estimates used in this study were sourced directly from the AIHW 2024 Burden of Disease Database, which includes pre-calculated risk attribution using established comparative risk assessment methodology. Additionally, we calculated sex- and age-standardized DALY rate, YLL rate, and YLD rate to estimate the contribution of sex and age to the NCDs burden.

### Ethics

Dataset created by AIHW is approved by the AIHW Ethics Committee. The evaluation process for the dataset includes a comprehensive assessment of potential impacts on individual privacy. Because the data used are secondary, aggregate-level data that do not involve identification of individuals, no additional Human Research Ethics Committee approval is required and no informed consent from participants was required. The research team commits to rigorously safeguarding data security and stakeholder interests throughout data analysis, report writing, and results dissemination. All outputs will be published in accordance with the AIHW Open Access Policy.

### Definitions

YLLs represents the years lost due to premature death, measuring the impact of diseases or injuries on population health by assessing the loss of life expectancy caused by different diseases. YLDs represents the years lived with disability, measuring the decline in quality of life due to diseases or injuries. It evaluates the impact of various diseases on an individual's daily life. DALYs is the sum of YLDs and YLLs, which is used to assess the overall burden of a disease or health condition on society. Attributable DALY/YLL/YLD/deaths means number of (linked disease) DALY/YLL/YLD/deaths attributable to the risk factor. Attributable DALY/YLL/YLD/deaths age-standardized rate (ASR) means the age-standardized rate of DALY/YLL/YLD/deaths attributable to the risk factor. All rates were age-standardized to the 2001 Australian Standard Population and are expressed per 1000 persons. Total DALY/YLL/YLD/deaths means the sum of all DALY/YLL/YLD/deaths.

### Data processing and analysis workflow

**Fig. 1:**
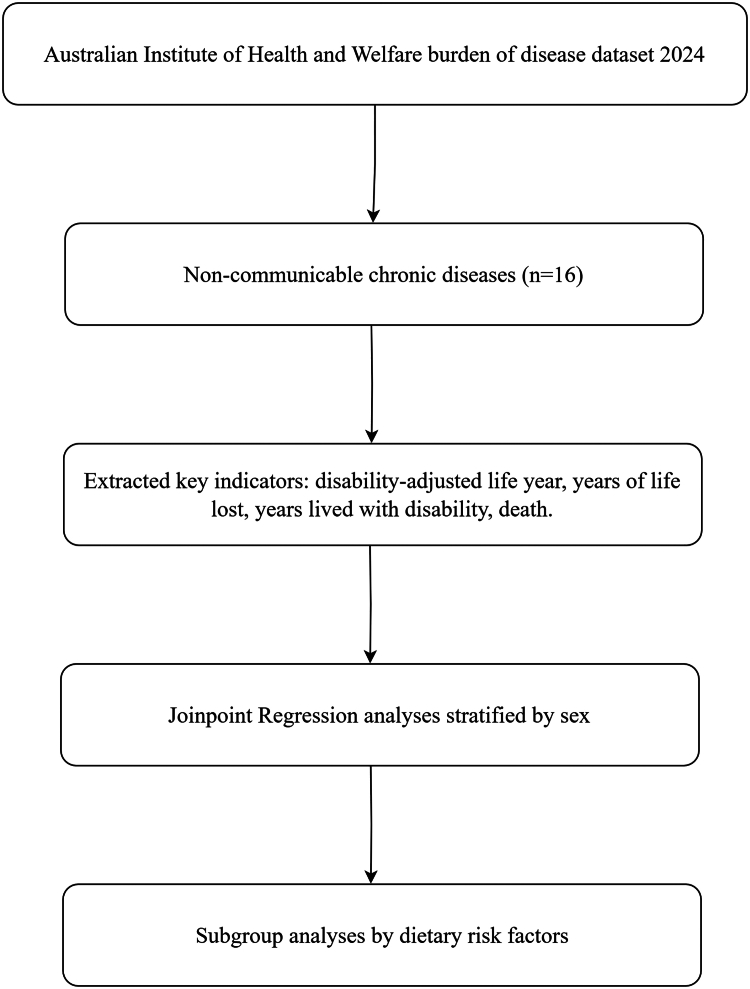
**Statistical analysis workflow**. N refers to the number of non-communicable chronic diseases.

The overall workflow of data processing and statistical analyses is summarized in Fig. 1. Briefly, we began by retrieving the AIHW 2024 burden of disease dataset and selecting all non-communicable chronic diseases of interest. For each disease, we extracted key indicators — DALYs, YLLs, YLDs, and deaths — and then performed Joinpoint Regression to detect temporal trend changes. Subgroup analyses were conducted by sex, dietary risk factor, and disease type to explore heterogeneity in trends across different subgroups and exposures.

### Statistics

The burden of NCD in Australia was assessed using annual deaths, DALYs, YLLs, and YLDs, and corresponding ASR. Rate differences between years and sex were assessed using two-sided t-tests. The standardized DALYs, YLLs, and YLDs were calculated based on both total and dietary-attributable burdens, depending on the analysis section. Specifically, all time trend analyses related to dietary risks were derived from risk-attributable DALYs and total DALYs. We calculated the Australia NCD-related age ASR, and the formula calculating ASR is as follows:

(ωi = Standard population in age group i, ri = Crude rate in age group i, Σ = Summation across all age groups. The result is multiplied by 1000 to express it per 1000 people.)

We analyzed the percentage change of deaths, DALYs, YLLs, and YLDs and the corresponding ASR from 2003 to 2024 to assess the trend of NCDs-related burden over the 21-year period. We included all estimates in the 95% uncertainty interval (95% UI). Annual Percentage Change (APC) was estimated using Joinpoint Regression analysis to determine statistically significant trends in dietary risk-attributable deaths and DALYs. The official website of the Joinpoint Regression Program developed by the U.S. National Cancer Institute (NCI): https://surveillance.cancer.gov/joinpoint. For each trend segment, APC was calculated as:

(β is the slope coefficient from the log-linear model. A positive APC indicates an increasing trend, and a negative APC indicates a decreasing trend. All estimates were reported with 95% confidence intervals (CI) and corresponding *P*-values.)

Stratified analyses were conducted by sex to evaluate disparities in disease burden over time. SAS JMP program (version 18.0) was used for all data analysis in this study. *P* < 0.05 was considered statistically significant.

### Role of funding source

This study was funded by the Australian Institute of Health and Welfare. The funder of the study had no role in study design, data collection, data analysis, data interpretation, or writing of this report.

## Results

### Overall status and trends in the burden of non-communicable chronic diseases from 2003 to 2024

This study included 16 diseases in the analysis of disease burden attributed to dietary risks, covering four major categories: cancer and other neoplasms, including bowel cancer, breast cancer, lung cancer, and oesophageal cancer; CVDs, including aortic aneurysm, atrial fibrillation and flutter (AF/AFL), cardiomyopathy, coronary heart disease (CHD), hypertensive heart disease (HHD), inflammatory heart disease, non-rheumatic valvular disease, peripheral vascular disease, rheumatic heart disease (including acute rheumatic fever), and stroke; endocrine disorders, represented by T2DM; and kidney and urinary diseases, represented by chronic kidney disease (CKD) (Table 1).

**Table 1:** Trends in the burden of disease attributable to dietary risk factors for non-communicable diseases in Australia from 2003 to 2024.

	Deaths					Attributable DALY				
	2003	2018	2024	2003–2018	2003–2024	2003	2018	2024	2003–2018	2003–2024
	Percentage, % (n)	Percentage, % (n)	Percentage, %	Percentage change, %	Percentage change, %	Percentage, % (n)	Percentage, % (n)	Percentage, % (n)	Percentage change, %	Percentage change, %
**Cancer and other neoplasms**										
Bowel cancer	24.6% (1298)	25.8% (1460)	25.7%	4.88%	4.47%	25.2% (24,767)	26.3% (25,942)	26.1% (25,579)	4.37%	3.57%
Breast cancer	3.0% (84)	3.2% (101)	3.2%	6.67%	6.67%	3.1% (2206)	3.3% (2352)	3.3% (2403)	6.45%	6.45%
Lung cancer	3.7% (269)	3.6% (330)	3.6%	−2.70%	−2.70%	3.7% (5171)	3.7% (5977)	3.7% (5855)	0	0
Oesophageal cancer	21.6% (252)	22.5% (311)	22.4%	4.17%	3.70%	22.0% (4854)	22.8% (5594)	22.7% (6078)	3.64%	3.18%
**Cardiovascular diseases**										
Aortic aneurysm	5.6% (76)	5.5% (57)	5.3%	−1.79%	−5.36%	6.2% (1137)	6.5% (931)	6.2% (982)	4.84%	0
Atrial fibrillation and flutter	5.0% (48)	4.4% (104)	4.5%	−12.00%	−10.00%	6.4% (1889)	6.0% (3486)	5.9% (4189)	−6.25%	−7.81%
Cardiomyopathy	6.3% (54)	6.0% (71)	6.0%	−4.76%	−4.76%	6.5% (1373)	6.5% (1691)	6.5% (1797)	0	0
Coronary heart disease	47.8% (13,461)	46.8% (9794)	46.4%	−2.09%	−2.93%	51.5% (219,047)	50.6% (158,800)	49.9% (158,265)	−1.75%	−3.11%
Hypertensive heart disease	6.4% (44)	6.0% (77)	6.0%	−6.25%	−6.25%	7.5% (554)	7.3% (920)	7.3% (1205)	−2.67%	−2.67%
Inflammatory heart disease	1.2% (2)	1.2% (4)	1.2%	0	0	1.1% (49)	1.2% (101)	1.3% (118)	9.09%	18.18%
Non-rheumatic valvular disease	4.1% (51)	3.8% (79)	3.8%	−7.32%	−7.32%	4.4% (1059)	4.3% (1305)	4.2% (1488)	−2.27%	−4.55%
Peripheral vascular disease	4.3% (36)	4.1% (34)	4.0%	−4.65%	−6.98%	4.7% (496)	4.7% (468)	4.7% (493)	0	0
Rheumatic heart disease (including acute rheumatic fever)	4.4% (14)	4.0% (15)	4.1%	−9.09%	−6.82%	4.6% (265)	4.6% (270)	4.5% (260)	0	−2.17%
Stroke	21.0% (2689)	21.7% (2315)	21.4%	3.33%	1.90%	24.3% (36,530)	25.7% (32,223)	25.0% (31,300)	5.76%	2.88%
**Endocrine disorders**										
Type 2 diabetes mellitus	24.4% (692)	25.5% (869)	25.4%	4.51%	4.10%	25.4% (19,671)	26.0% (29,170)	26.0% (33,129)	2.36%	2.36%
**Kidney and urinary diseases**										
Chronic kidney disease	6.4% (134)	6.2% (201)	6.2%	−3.13%	−3.13%	6.7% (2229)	6.6% (3247)	6.6% (4285)	−1.49%	−1.49%
Total	27.87% (19,204)	23.61% (15,822)		−15.29%		28.23% (321,297)	24.28% (272,477)	23.45% (277,426)	−13.99%	−16.93%

	Attributable YLL					Attributable YLD				
	2003	2018	2024	2003–2018	2003–2024	2003	2018	2024	2003–2018	2003–2024
	Percentage, % (n)	Percentage, % (n)	Percentage, % (n)	Percentage change, %	Percentage change, %	Percentage, % (n)	Percentage, % (n)	Percentage, % (n)	Percentage change, %	Percentage change, %
**Cancer and other neoplasms**										
Bowel cancer	25.2% (23,456)	26.3% (24,264)	26.2% (23,762)	4.37%	3.97%	24.6% (1311)	25.8% (1678)	25.8% (1817)	4.88%	4.88%
Breast cancer	3.1% (1995)	3.3% (2032)	3.3% (2036)	6.45%	6.45%	3.0% (211)	3.3% (320)	3.3% (367)	10.00%	10.00%
Lung cancer	3.7% (5085)	3.7% (5853)	3.7% (5709)	0	0	3.6% (85)	3.6% (124)	3.6% (147)	0	0
Oesophageal cancer	22.0% (4767)	22.8% (5475)	22.7% (5942)	3.64%	3.18%	21.6% (87)	22.4% (119)	22.4% (136)	3.70%	3.70%
**Cardiovascular diseases**										
Aortic aneurysm	6.2% (1136)	6.5% (931)	6.2% (981)	4.84%	0	6.7% (1)	6.7% (1)	6.5% (0)	0	−2.99%
Atrial fibrillation and flutter	5.6% (458)	5.0% (828)	5.1% (1063)	−10.71%	−8.93%	6.7% (1431)	6.3% (2658)	6.3% (3126)	−5.97%	−5.97%
Cardiomyopathy	6.6% (1218)	6.6% (1492)	6.7% (1565)	0	1.52%	5.8% (155)	5.8% (199)	5.7% (232)	0	−1.72%
Coronary heart disease	51.7% (180,738)	51.0% (125,067)	50.3% (125,405)	−1.35%	−2.71%	50.6% (38,309)	49.0% (33,733)	48.5% (32,860)	−3.16%	−4.15%
Hypertensive heart disease	7.5% (545)	7.3% (909)	7.3% (1193)	−2.67%	−2.67%	6.4% (9)	6.2% (11)	6.1% (13)	−3.13%	−4.69%
Inflammatory heart disease	1.1% (36)	1.3% (84)	1.3% (99)	18.18%	18.18%	1.1% (13)	1.1% (17)	1.1% (19)	0	0
Non-rheumatic valvular disease	4.4% (615)	4.3% (803)	4.2% (914)	−2.27%	−4.55%	4.3% (444)	4.2% (502)	4.2% (574)	−2.33%	−2.33%
Peripheral vascular disease	4.6% (398)	4.7% (389)	4.6% (417)	2.17%	0	5.1% (98)	5.0% (78)	4.9% (76)	−1.96%	−3.92%
Rheumatic heart disease (including acute rheumatic fever)	4.6% (228)	4.6% (235)	4.5% (225)	0	−2.17%	4.5% (36)	4.4% (35)	4.4% (35)	−2.22%	−2.22%
Stroke	24.2% (33,079)	25.7% (27,872)	25.0% (26,612)	6.20%	3.31%	24.4% (3451)	26.0% (4351)	25.3% (4688)	6.56%	3.69%
**Endocrine disorders**										
Type 2 diabetes mellitus	25.1% (9897)	25.9% (11,388)	25.8% (12,562)	3.19%	2.79%	25.8% (9774)	26.1% (17,783)	26.0% (20,567)	1.16%	0.78%
**Kidney and urinary diseases**										
Chronic kidney disease	6.7% (1636)	6.6% (2286)	6.6% (3100)	−1.49%	−1.49%	6.6% (593)	6.6% (960)	6.6% (1185)	0	0
Total	27.99% (265,287)	24.06% (209,908)	23.38% (211,585)	−14.04%	−16.47%	29.44% (56,008)	25.04% (62,569)	23.68% (65,842)	−14.95%	−19.57%

N refers to the number of deaths, DALYs, YLLs, or YLDs; % represents the proportion attributable to all dietary risk factors.

Abbreviations: Attributable DALY, number of (linked disease) DALYs attributable to the risk factor. DALY, disability-adjusted life year. Attributable YLL, number of (linked disease) YLLs attributable to the risk factor. YLL, years of life lost. Attributable YLD, number of (linked disease) YLDs attributable to the risk factor. YLD, years lived with disability.

In NCDs, the overall burden of disease attributed to dietary risks has decreased. The number of deaths attributed to dietary risks decreased from 27.87% (19,204) in 2003 to 23.61% (15,822) in 2018, marking a 15.29% reduction over the 15-year study period. From 2003 to 2024, the most significant decrease in deaths was recorded for AF/AFL (−10.00%). By comparison, from 2003 to 2024, the most significant increase in deaths was seen in breast cancer (6.67%), followed by bowel cancer (4.47%). The overall trend in DALYs attributed to dietary risks showed a similar decline, dropping from 28.23% (321,297) in 2003 to 23.45% (277,426) in 2024, with a 16.93% reduction over the 21-year study period. The most notable decrease in DALYs was seen in AF/AFL (−7.81%). Conversely, the most significant increase in DALYs was observed in inflammatory heart disease (18.18%). The total number of YLLs attributed to dietary risks decreased from 27.99% (265,287) in 2003 to 23.38% (211,585) in 2024, marking a 16.47% reduction over the 21-year study period. The most notable decrease in YLLs was seen in AF/AFL (−8.93%). Conversely, the most significant increase in YLLs was observed in inflammatory heart disease (18.18%). The total number of YLDs attributed to dietary risks decreased from 29.44% (56,008) in 2003 to 23.68% (65,842) in 2024, marking a 19.57% reduction over the 21-year study period. The most notable decrease in YLDs was seen in AF/AFL (−5.97%). Conversely, the most significant increase in YLDs was observed in breast cancer (10.00%).

### The age-standardized rate in the disability-adjusted life years of non-communicable chronic diseases by sex subgroup from 2003 to 2024

From 2003 to 2024, the ASR in DALY for most NCDs mainly showed decline trends (Supplementary Table S1).

Among cancers and other neoplasms, bowel cancer had the largest decline, with ASR dropping from 3.624 per 1000 population (95% UI: 3.600, 3.647) in 2003 to 2.151 per 1000 population (95% UI: 2.136, 2.165) in 2024, corresponding to an APC of −2.360% (95% CI: −2.975%, −1.719%, *P* < 0.001) in persons. Among CVDs, the greatest decline in persons was observed in CHD, with the ASR dropping from 14.166 per 1000 population (95% UI: 14.122, 14.210) in 2003 to 6.123 per 1000 population (95% UI: 6.100, 6.147) in 2024. This corresponds to an APC of −4.016% (95% CI: −4.578%, −3.517%, *P* < 0.001). In contrast, the most significant increase was seen in AF/AFL, rising from 0.954 per 1000 population (95% UI: 0.943, 0.965) in 2003 to 1.207 per 1000 population (95% UI: 1.197, 1.217) in 2024, with an APC of 1.170% (95% CI: 0.384%, 2.132%, *P* < 0.001). In T2DM, only males experienced the a statistically significant decline, decreasing from 3.508 per 1000 population (95% UI: 3.475, 3.540) in 2003 to 3.373 per 1000 population (95% UI: 3.348, 3.398) in 2024, with an APC of −0.178% (−0.220%, −0.135%, *P* < 0.001). The trend in CKD remained relatively stable, showing no significant change over the study period.

**Fig. 2:**
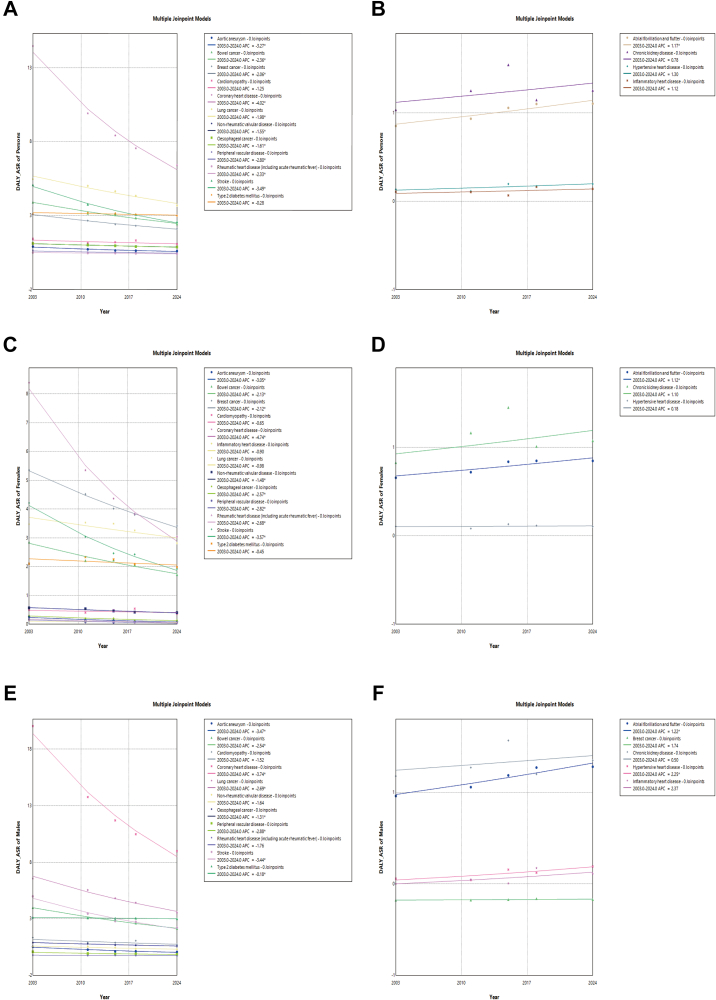
**Trends in age-standardized disability-adjusted life year rates (DALY-ASR) based on joinpoint regression for non-communicable diseases from 2003 to 2024 by sex**. A: DALY-ASR of persons decreased. B: DALY-ASR of persons increased. C: DALY-ASR of females decreased. D: DALY-ASR of females increased. E: DALY-ASR of males decreased. F: DALY-ASR of males increased.

Subgroup analysis by sex was performed using Joinpoint Regression on DALYs for all NCDs. In the total population, CHD showed the most consistent and statistically significant decreasing trend, with an APC of −4.02% (95% CI: −4.58%, −3.52%) (Fig. 2A); in contrast, AF/AFL exhibited the most consistent and statistically significant increasing trend, with an APC of 1.17% (95% CI: 0.38%, 2.13%) (Fig. 2B). In females, CHD demonstrated a statistically significant linear decline (APC = −4.74%, 95% CI: −5.49%, −4.06%) (Fig. 2C), and AF/AFL showed a significant upward trend (APC = 1.12%, 95% CI: 0.27%, 2.12%) (Fig. 2D). In males, CHD also declined a consistent and statistically significant decline (APC = −3.74%, 95% CI: −4.28%, −3.25%) (Fig. 2E), while HHD showed a significant increasing trend (APC = 2.25%, 95% CI: 0.43%, 4.65%) (Fig. 2F).

### The age-standardized rate in the years of life lost of non-communicable chronic diseases by sex subgroup from 2003 to 2024

From 2003 to 2024, the ASR in YLL for most NCDs mainly showed decline trends (Supplementary Table S2).

In cancer and other neoplasms, the ASR in YLL demonstrated a declining trend among persons, with bowel cancer showing the most significant decrease, from 3.444 per 1000 population (95% UI: 3.422, 3.467) in 2003 to 2.018 per 1000 population (95% UI: 2.004, 2.032) in 2024, with an APC of −2.414% (95% CI: −3.062%, −1.741%, *P* < 0.001). In CVDs, the ASR demonstrated a declining trend among persons, with CHD showing the most significant decrease, from 11.683 per 1000 population (95% UI: 11.643, 11.723) in 2003 to 4.900 per 1000 population (95% UI: 4.879, 4.921) in 2024, with an APC of −4.180% (95% CI: −4.820%, −3.614%, *P* < 0.001). T2DM decreased among persons from 1.366 per 1000 population (95% UI: 1.352, 1.380) in 2003 to 0.938 per 1000 population (95% UI: 0.930, 0.947) in 2024, with an APC of −1.797% (95% CI: −2.979%, −0.508%, *P* < 0.001). The trend in CKD remained relatively stable, showing no statistically significant change over the study period.

**Fig. 3:**
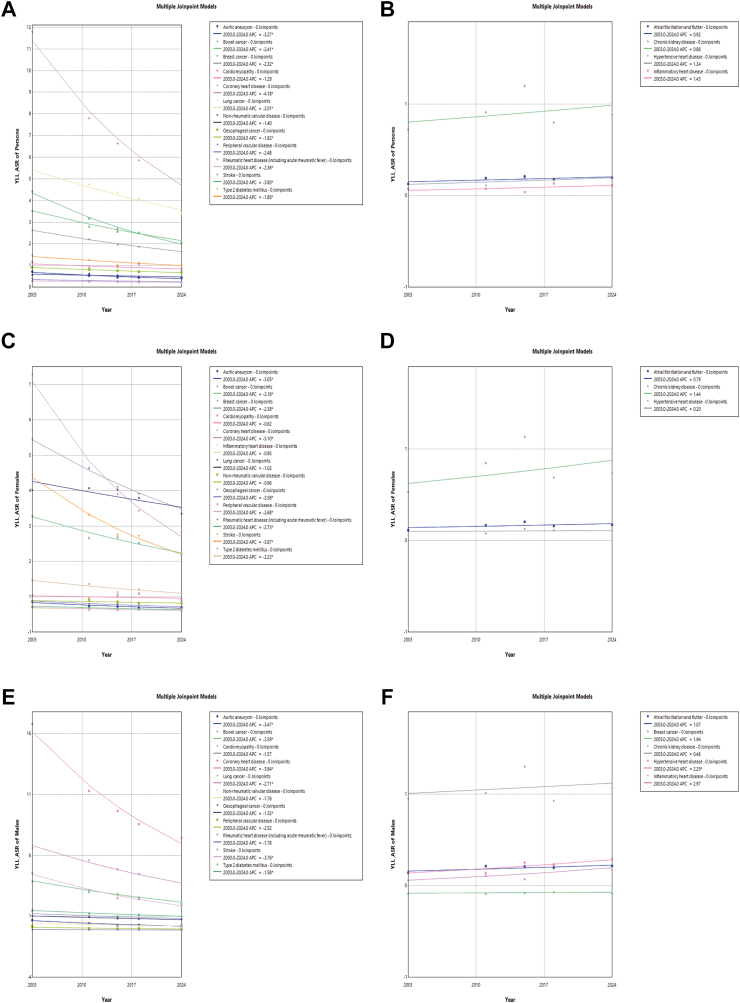
**Trends in age-standardized years of life lost rates (YLL-ASR) based on Joinpoint Regression for non-communicable diseases from 2003 to 2024 by sex**. A: YLL-ASR of persons decreased. B: YLL-ASR of persons increased. C: YLL-ASR of females decreased. D: YLL-ASR of females increased. E: YLL-ASR of males decreased. F: YLL-ASR of males increased.

Subgroup analysis by sex was performed using Joinpoint Regression on YLLs for all NCDs. Among individuals, CHD showed a significant decreasing trend, with an APC of −4.18% (95% CI: −4.82% to −3.61%) (Fig. 3A), whereas none of the increasing trends in Fig. 3B were statistically significant. In females, CHD showed a significant decreasing trend, with an APC of −5.10% (95% CI: −5.95% to −4.36%) (Fig. 3C), while none of the increasing trends were statistically significant (Fig. 3D). In males, CHD also significantly declined, with an APC of −3.84% (95% CI: −4.42% to −3.31%) (Fig. 3E). In contrast, HHD showed a significant increasing trend in males, with an APC of 2.29% (95% CI: 0.50%–4.66%) (Fig. 3F).

### The age-standardized rate in the years lived with disability lost of non-communicable chronic diseases by sex subgroup from 2003 to 2024

From 2003 to 2024, the ASR in YLD for most NCDs showed varying trends (Supplementary Table S3).

In cancer and other neoplasms, the ASR showed decline trends. Among persons, Bowel cancer decreased most significantly from 0.179 per 1000 population (95% UI: 0.174, 0.184) in 2003 to 0.133 per 1000 population (95% UI: 0.130, 0.136) in 2024, with an APC of −1.437% (−2.102%, −0.708%, *P* < 0.001). Notably, Breast cancer in males showed a statistically increase from 0.004 per 1000 population (95% UI: 0.003, 0.005) in 2003 to 0.005 per 1000 population (95% UI: 0.004, 0.006) in 2024, with an APC of 1.092% (95% CI: 0.324%, 1.948%, *P* = 0.004). Lung cancer in females showed a statistically increase from 0.053 per 1000 population (95% UI: 0.049, 0.057) in 2003 to 0.068 per 1000 population (95% UI: 0.064, 0.071) in 2024, with an APC of 1.109% (95% CI: 0.751%, 1.541%, *P* < 0.001). In CVDs, aortic aneurysm showed the most significant decline in the total population and males. Among persons, the ASR of YLD decreased from 0.001 per 1000 population (95% UI: 0, 0.001) in 2003 to 0 per 1000 population (95% UI: 0, 0) in 2024, with an APC of −8.416% (95% CI: −9.294%, −7.720%, *P* < 0.001). In CKD, the ASR rose from 0.310 per 1000 population (95% UI: 0.303, 0.316) in 2003 to 0.359 per 1000 population (95% UI: 0.353, 0.365) in 2024, with an APC of 0.653% (95% CI: 0.298%, 1.064%, *P* < 0.001). The trend in T2DM remained relatively stable, showing no statistically significant change over the study period.

**Fig. 4:**
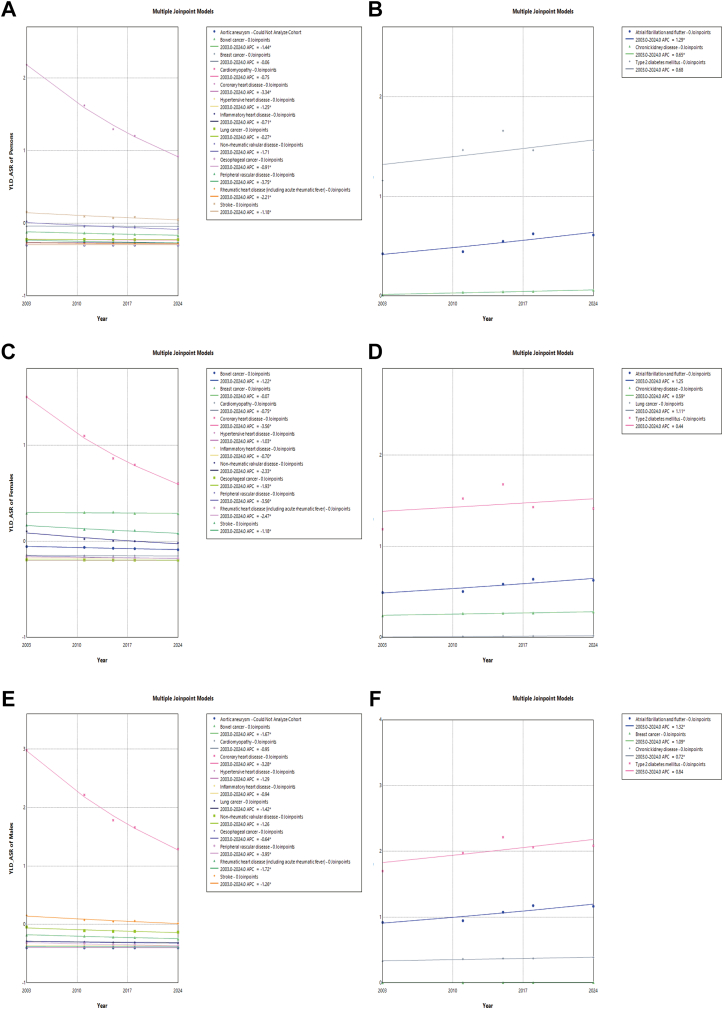
**Trends in age-standardized years lived with disability rates (YLD-ASR) based on Joinpoint Regression for non-communicable diseases from 2003 to 2024 by sex**. A: YLD-ASR of persons decreased. B: YLD-ASR of persons increased. C: YLD-ASR of females decreased. D: YLD-ASR of females increased. E: YLD-ASR of males decreased. F: YLD-ASR of males increased.

Subgroup analysis by sex was performed using Joinpoint Regression on YLDs for all NCDs. Aortic aneurysm showed a significant decreasing trend among the population, with an APC of −8.42% (95% CI: −9.29% to −7.72%) (Fig. 4A). In contrast, AF/AFL and flutter significantly increased, with an APC of 1.29% (95% CI: 0.001%–2.92%) (Fig. 4B). In females, CHD and peripheral vascular disease both showed significant declines, with APC of −3.56% (95% CI: −3.85% to −3.28%) and −3.56% (95% CI: −4.76% to −2.43%), respectively (Fig. 4C); in contrast, lung cancer showed a significant increase, with an APC of 1.11% (95% CI: 0.75%–1.54%) (Fig. 4D). In males, aortic aneurysm showed a significant decreasing trend, with an APC of −8.30% (95% CI: −9.15% to −7.65%) (Fig. 4E), while AF/AFL and flutter showed a significant increase, with an APC of 1.32% (95% CI: 0.04%–2.95%) (Fig. 4F).

### Dietary risk-attributable disability-adjusted life years from non-communicable chronic diseases in Australia from 2003 to 2024

The research examined 13 dietary risk factors attributed to NCDs from 2003 to 2024, including: all dietary risks, diet high in processed meat, diet high in red meat, diet high in sodium, diet high in sugar-sweetened beverages, diet low in fruit, diet low in legumes, diet low in milk, diet low in nuts and seeds, diet low in polyunsaturated fats, diet low in vegetables, diet low in whole grains and high fibre cereals. The percentage change in DALYs was used to compare the impact of dietary risk factors from 2003 to 2024, aiming to identify the primary dietary risk factors to the burden of NCDs (Table 2).

**Table 2:** DALYs attributable to dietary risk factors for non-communicable diseases in Australia from 2003 to 2024.

	Risk Factors	Sex	2003	Total DALY (n)	2024	Total DALY (n)	2023–2024
			Attributable DALY percentage, % (n)		Attributable DALY percentage, % (n)		Percentage change, %
**Cancer and other neoplasms**							
Bowel cancer							
	All dietary risks	Female	25.1% (10,668)	42,454	26.1% (11,266)	43,180	3.98%
		Male	25.2% (14,099)	55,948	26.2% (14,312)	54,723	3.97%
		Person	25.2% (24,767)	98,403	26.1% (25,579)	97,903	3.57%
	Diet high in processed meat	Female	2.1% (873)	42,454	2.2% (967)	43,180	4.76%
		Male	2.1% (1149)	55,948	2.2% (1213)	54,723	4.76%
		Person	2.1% (2022)	98,403	2.2% (2180)	97,903	4.76%
	Diet high in red meat	Female	5.3% (2251)	42,454	5.8% (2495)	43,180	9.43%
		Male	5.4% (2998)	55,948	5.8% (3182)	54,723	7.41%
		Person	5.3% (5249)	98,403	5.8% (5677)	97,903	9.43%
	Diet low in milk	Female	4.5% (1896)	42,454	4.7% (2027)	43,180	4.44%
		Male	4.5% (2516)	55,948	4.7% (2578)	54,723	4.44%
		Person	4.5% (4412)	98,403	4.7% (4605)	97,903	4.44%
	Diet low in whole grains & high fibre cereals	Female	15.5% (6589)	42,454	15.8% (6831)	43,180	1.94%
		Male	15.5% (8683)	55,948	15.9% (8680)	54,723	2.58%
		Person	15.5% (15,271)	98,403	15.8% (15,510)	97,903	1.94%
Breast cancer							
	All dietary risks	Female	3.1% (2198)	70,527	3.3% (2382)	72,659	6.45%
		Male	3.0% (8)	277	3.2% (21)	650	6.67%
		Person	3.1% (2206)	70,804	3.3% (2403)	73,309	6.45%
	Diet high in red meat	Female	3.1% (2198)	70,527	3.3% (2382)	72,659	6.45%
		Male	3.0% (8)	277	3.2% (21)	650	6.67%
		Person	3.1% (2206)	70,804	3.3% (2403)	73,309	6.45%
Lung cancer							
	All dietary risks	Female	3.8% (1868)	49,803	3.7% (2566)	69,384	−2.63%
		Male	3.7% (3302)	88,909	3.7% (3290)	89,061	0
		Person	3.7% (5171)	138,712	3.7% (5855)	158,445	0
	Diet low in fruit	Female	3.8% (1868)	49,803	3.7% (2566)	69,384	−2.63%
		Male	3.7% (3302)	88,909	3.7% (3290)	89,061	0
		Person	3.7% (5171)	138,712	3.7% (5855)	158,445	0
Oesophageal cancer							
	All dietary risks	Female	21.9% (1293)	5911	22.6% (1354)	6001	3.20%
		Male	22.1% (3561)	16,140	22.7% (4724)	20,767	2.71%
		Person	22.0% (4854)	22,051	22.7% (6078)	26,768	3.18%
	Diet low in fruit	Female	16.1% (950)	5911	16.0% (960)	6001	−0.62%
		Male	16.4% (2649)	16,140	16.3% (3384)	20,767	−0.61%
		Person	16.3% (3599)	22,051	16.2% (4344)	26,768	−0.61%
	Diet low in vegetables	Female	6.9% (409)	5911	7.8% (468)	6001	13.04%
		Male	6.8% (1091)	16,140	7.7% (1600)	20,767	13.24%
		Person	6.8% (1500)	22,051	7.7% (2068)	26,768	13.24%
**Cardiovascular diseases**							
Aortic aneurysm							
	All dietary	Female	4.8% (293)	6155	4.5% (252)	5587	−6.25%
		Male	7.0% (844)	12,071	7.2% (730)	10,127	2.86%
		Person	6.2% (1137)	18,227	6.2% (982)	15,714	0
	Diet high in sodium	Female	4.8% (293)	6155	4.5% (252)	5587	−6.25%
		Male	7.0% (844)	12,071	7.2% (730)	10,127	2.86%
		Person	6.2% (1137)	18,227	6.2% (982)	15,714	0
Atrial fibrillation and flutter							
	All dietary risks	Female	5.1% (732)	14,282	4.4% (1441)	32,647	−13.73%
		Male	7.5% (1157)	15,471	7.2% (2747)	38,153	−4.00%
		Person	6.4% (1889)	29,753	5.9% (4189)	70,799	−7.81%
	Diet high in sodium	Female	5.1% (732)	14,282	4.4% (1441)	32,647	−13.73%
		Male	7.5% (1157)	15,471	7.2% (2747)	38,153	−4.00%
		Person	6.4% (1889)	29,753	5.9% (4189)	70,799	−7.81%
Cardiomyopathy							
	All dietary risks	Female	4.5% (266)	5909	4.5% (380)	8445	0
		Male	7.2% (1107)	15,327	7.4% (1417)	19,185	2.78%
		Person	6.5% (1373)	21,236	6.5% (1797)	27,630	0
	Diet high in sodium	Female	4.5% (266)	5909	4.5% (380)	8445	0
		Male	7.2% (1107)	15,327	7.4% (1417)	19,185	2.78%
		Person	6.5% (1373)	21,236	6.5% (1797)	27,630	0
Coronary heart disease							
	All dietary risks	Female	48.2% (76,426)	158,641	46.8% (47,004)	100,524	−2.90%
		Male	53.5% (142,621)	266,458	51.4% (111,261)	216,581	−3.93%
		Person	51.5% (219,047)	425,099	49.9% (158,265)	317,105	−3.11%
	Diet high in processed meat	Female	1.9% (2965)	158,641	2.2% (2168)	100,524	15.79%
		Male	2.3% (6110)	266,458	2.4% (5147)	216,581	4.35%
		Person	2.1% (9075)	425,099	2.3% (7315)	317,105	9.52%
	Diet high in red meat	Female	4.4% (6928)	158,641	5.0% (4994)	100,524	13.64%
		Male	5.3% (14,008)	266,458	5.6% (12,096)	216,581	5.66%
		Person	4.9% (20,935)	425,099	5.4% (17,090)	317,105	10.20%
	Diet high in sodium	Female	6.1% (9598)	158,641	5.4% (5470)	100,524	−11.48%
		Male	8.7% (23,272)	266,458	8.8% (19,054)	216,581	1.15%
		Person	7.7% (32,870)	425,099	7.7% (24,524)	317,105	0
	Diet high in sugar-sweetened beverages	Female	0.5% (759)	158,641	0.5% (484)	100,524	0
		Male	1.1% (2869)	266,458	1.0% (2199)	216,581	−9.09%
		Person	0.9% (3628)	425,099	0.8% (2683)	317,105	−11.11%
	Diet low in fish & seafood	Female	2.0% (3112)	158,641	1.6% (1572)	100,524	−20.00%
		Male	2.3% (6186)	266,458	1.7% (3733)	216,581	−26.09%
		Person	2.2% (9298)	425,099	1.7% (5305)	317,105	−22.73%
	Diet low in fruit	Female	4.7% (7412)	158,641	4.7% (4682)	100,524	0
		Male	5.3% (14,244)	266,458	5.2% (11,163)	216,581	−1.89%
		Person	5.1% (21,656)	425,099	5.0% (15,846)	317,105	−1.96%
	Diet low in legumes	Female	18.7% (29,611)	158,641	17.7% (17,841)	100,524	−5.35%
		Male	20.9% (55,684)	266,458	19.4% (42,055)	216,581	−7.18%
		Person	20.1% (85,295)	425,099	18.9% (59,896)	317,105	−5.97%
	Diet low in nuts & seeds	Female	12.0% (19,025)	158,641	9.7% (9785)	100,524	−19.17%
		Male	13.2% (35,159)	266,458	10.1% (21,961)	216,581	−23.48%
		Person	12.7% (54,183)	425,099	10.0% (31,746)	317,105	−21.26%
	Diet low in polyunsaturated fats	Female	1.6% (2459)	158,641	1.6% (1566)	100,524	0
		Male	1.9% (5028)	266,458	1.8% (4003)	216,581	−5.26%
		Person	1.8% (7487)	425,099	1.8% (5568)	317,105	0
	Diet low in vegetables	Female	5.1% (8053)	158,641	5.8% (5816)	100,524	13.73%
		Male	5.5% (14,600)	266,458	6.1% (13,253)	216,581	10.91%
		Person	5.3% (22,653)	425,099	6.0% (19,069)	317,105	13.21%
	Diet low in whole grains & high fibre cereals	Female	6.4% (10,113)	158,641	6.6% (6652)	100,524	3.13%
		Male	7.4% (19,747)	266,458	7.4% (16,015)	216,581	0
		Person	7.0% (29,860)	425,099	7.1% (22,667)	317,105	1.43%
Hypertensive heart disease							
	All dietary risks	Female	6.1% (247)	4066	5.3% (400)	7502	−13.11%
		Male	9.2% (307)	3322	9.0% (805)	8943	−2.17%
		Person	7.5% (554)	7388	7.3% (1205)	16,445	−2.67%
	Diet high in sodium	Female	6.1% (247)	4066	5.3% (400)	7502	−13.11%
		Male	9.2% (307)	3322	9.0% (805)	8943	−2.17%
		Person	7.5% (554)	7388	7.3% (1205)	16,445	−2.67%
Inflammatory heart disease							
	All dietary risks	Female	0.9% (19)	2058	0.9% (33)	3512	0
		Male	1.3% (31)	2356	1.5% (85)	5597	15.38%
		Person	1.1% (49)	4413	1.3% (118)	9109	18.18%
	Diet high in sodium	Female	0.9% (19)	2058	0.9% (33)	3512	0
		Male	1.3% (31)	2356	1.5% (85)	5597	15.38%
		Person	1.1% (49)	4413	1.3% (118)	9109	18.18%
Non-rheumatic valvular disease							
	All dietary risks	Female	3.7% (449)	12,157	3.2% (562)	17,364	−13.51%
		Male	5.1% (610)	11,998	5.1% (927)	18,086	0
		Person	4.4% (1059)	24,154	4.2% (1488)	35,450	−4.55%
	Diet high in sodium	Female	3.7% (449)	12,157	3.2% (562)	17,364	−13.51%
		Male	5.1% (610)	11,998	5.1% (927)	18,086	0
		Person	4.4% (1059)	24,154	4.2% (1488)	35,450	−4.55%
Peripheral vascular disease							
	All dietary risks	Female	3.9% (198)	5035	3.5% (155)	4487	−10.26%
		Male	5.4% (298)	5559	5.6% (338)	6066	3.70%
		Person	4.7% (496)	10,594	4.7% (493)	10,552	0
	Diet high in sodium	Female	3.9% (198)	5035	3.5% (155)	4487	−10.26%
		Male	5.4% (298)	5559	5.6% (338)	6066	3.70%
		Person	4.7% (496)	10,594	4.7% (493)	10,552	0
Rheumatic heart disease							
	All dietary risks	Female	3.9% (147)	3746	3.7% (133)	3551	−5.13%
		Male	5.9% (118)	1985	5.7% (128)	2232	−3.39%
		Person	4.6% (265)	5731	4.5% (260)	5783	−2.17%
	Diet high in sodium	Female	3.9% (147)	3746	3.7% (133)	3551	−5.13%
		Male	5.9% (118)	1985	5.7% (128)	2232	−3.39%
		Person	4.6% (265)	5731	4.5% (260)	5783	−2.17%
Stroke							
	All dietary risks	Female	22.9% (18,020)	78,853	23.4% (14,225)	60,897	2.18%
		Male	25.8% (18,510)	71,763	26.6% (17,075)	64,244	3.10%
		Person	24.3% (36,530)	150,616	25.0% (31,300)	125,141	2.88%
	Diet high in red meat	Female	6.6% (5195)	78,853	7.3% (4417)	60,897	10.61%
		Male	7.3% (5248)	71,763	7.7% (4972)	64,244	5.48%
		Person	6.9% (10,444)	150,616	7.5% (9390)	125,141	8.70%
	Diet high in sodium	Female	5.8% (4592)	78,853	5.3% (3235)	60,897	−8.62%
		Male	8.0% (5721)	71,763	8.2% (5282)	64,244	2.50%
		Person	6.8% (10,313)	150,616	6.8% (8517)	125,141	0
	Diet low in fruit	Female	4.8% (3778)	78,853	4.8% (2897)	60,897	0
		Male	5.2% (3734)	71,763	5.1% (3245)	64,244	−1.92%
		Person	5.0% (7511)	150,616	4.9% (6142)	125,141	−2.00%
	Diet low in vegetables	Female	5.9% (4621)	78,853	6.3% (3826)	60,897	6.78%
		Male	6.1% (4373)	71,763	6.5% (4154)	64,244	6.56%
		Person	6.0% (8995)	150,616	6.4% (7979)	125,141	6.67%
	Diet low in whole grains & high fibre cereals	Female	2.6% (2072)	78,853	2.7% (1656)	60,897	3.85%
		Male	2.9% (2090)	71,763	2.9% (1878)	64,244	0
		Person	2.8% (4162)	150,616	2.8% (3534)	125,141	0
**Endocrine disorders**							
Type 2 diabetes mellitus							
	All dietary risks	Female	24.7% (8049)	32,596	25.5% (13,284)	52,097	3.24%
		Male	25.9% (11,622)	44,860	26.3% (19,845)	75,496	1.54%
		Person	25.4% (19,671)	77,457	26.0% (33,129)	127,592	2.36%
	Diet high in processed meat	Female	5.2% (1691)	32,596	5.7% (2991)	52,097	9.62%
		Male	5.3% (2374)	44,860	5.7% (4314)	75,496	7.55%
		Person	5.2% (4065)	77,457	5.7% (7304)	127,592	9.62%
	Diet high in red meat	Female	7.8% (2528)	32,596	8.6% (4458)	52,097	10.26%
		Male	7.9% (3562)	44,860	8.6% (6489)	75,496	8.86%
		Person	7.9% (6090)	77,457	8.6% (10,947)	127,592	8.86%
	Diet high in sugar-sweetened beverages	Female	1.4% (450)	32,596	1.4% (703)	52,097	0
		Male	2.5% (1114)	44,860	2.3% (1772)	75,496	−8.00%
		Person	2.0% (1564)	77,457	1.9% (2476)	127,592	−5.00%
	Diet low in fruit	Female	6.5% (2130)	32,596	6.5% (3400)	52,097	0
		Male	6.6% (2976)	44,860	6.6% (4949)	75,496	0
		Person	6.6% (5106)	77,457	6.5% (8349)	127,592	−1.52%
	Diet low in nuts & seeds	Female	2.7% (866)	32,596	2.1% (1104)	52,097	−22.22%
		Male	2.7% (1197)	44,860	2.1% (1587)	75,496	−22.22%
		Person	2.7% (2063)	77,457	2.1% (2691)	127,592	−22.22%
	Diet low in whole grains & high fibre cereals	Female	4.1% (1337)	32,596	4.2% (2214)	52,097	2.44%
		Male	4.2% (1866)	44,860	4.3% (3223)	75,496	2.38%
		Person	4.1% (3203)	77,457	4.3% (5437)	127,592	4.88%
**Kidney and urinary diseases**							
Chronic kidney disease							
	All dietary risks	Female	5.7% (910)	16,009	5.1% (1563)	30,501	−10.53%
		Male	7.6% (1318)	17,395	7.9% (2721)	34,589	3.95%
		Person	6.7% (2229)	33,404	6.6% (4285)	65,089	−1.49%
	Diet high in sodium	Female	5.7% (910)	16,009	5.1% (1563)	30,501	−10.53%
		Male	7.6% (1318)	17,395	7.9% (2721)	34,589	3.95%
		Person	6.7% (2229)	33,404	6.6% (4285)	65,089	−1.49%

N refers to the number of DALYs; % represents the proportion of total DALYs attributable to each dietary risk factor.

Abbreviations: Attributable DALY, number of (linked disease) DALYs attributable to the risk factor. Total DALY, sum of all DALYs. DALY, disability-adjusted life year.

### Cancer and other neoplasms

Among persons, the impact of diet high in red meat on bowel cancer showed a more significant impact on females than on males, accounting from 5.3% (2251) in 2003 to 5.8% (2495) in 2024, percentage change 9.43%; from 5.4% (2998) in 2003 to 5.8% (3182) in 2024, percentage change 7.41%, respectively. For breast cancer, a diet high in red meat has a more significant impact on males than on females, from 3.0% (8) in 2003 to 3.2% (21) in 2024, representing a 6.67% increase; from 3.1% (2198) in 2003 to 3.3% (2382) in 2024, a percentage change of 6.45%, respectively. The impact of dietary low in fruit on lung cancer among women has a slight decline of 2.63%, from 3.8% (1868) in 2003 to 3.7% (2566) in 2024. Diet low in vegetables emerged as the main driver in oesophageal cancer, rising from 6.8% (1500) in 2003 to 7.7% (2068) in 2024 with an increased percentage change of 13.24%, which was observed in both males and females. In comparison, the proportion linked to a diet low in fruit slightly declined from 16.3% (3599) in 2003 to 16.2% (4344) in 2024, with a decreased percentage change of 0.61%.

### Cardiovascular diseases

CVDs with increased DALYs attributable to dietary risks were inflammatory heart disease and stroke. Between them, the largest increase was observed in inflammatory heart disease driven by diet high in sodium, with a rise of 18.18% (from 1.1%, 49 in 2003 to 1.3%, 118 in 2024). CVDs with declining DALYs attributable to dietary risks include aortic aneurysm, AF/AFL, cardiomyopathy, CHD, HHD, rheumatic heart disease, non-rheumatic valvular disease, and peripheral vascular disease. Among these, AF/AFL driven by diet high in sodium showed the greatest decrease with a 7.81% reduction, from 6.4% (1889) in 2003 to 5.9% (4189) in 2024. In females, the largest increase was diet high in processed meat contributing to DALY occurred in CHD, with a 15.79% rise (from 1.9%, 2965 in 2003 to 2.2%, 2168 in 2024); while the largest decrease was diet low in fish and seafood attributable DALY occurred in CHD, with a 20.00% decrease (from 2.0%, 3112 in 2003 to 1.6%, 1572 in 2024). In males, the largest increase in DALYs attributable to a diet low in vegetables was observed in inflammatory heart disease, with a 15.38% rise, from 1.3% (31) in 2003 to 1.5% (85) in 2024; the largest decrease in DALYs attributable to a diet low in fish and seafood in CHD was observed as a 26.09% reduction—from 2.3% (6186) in 2003 to 1.7% (3733) in 2024.

### Endocrine disorders

Overall, dietary risks attributable to DALYs for T2DM increased by 2.36%, rising from 25.4% (19,671) in 2003 to 26.0% (33,129) in 2024. Among these, diets high in processed meat and red meat were the two fastest-growing dietary risk factors. This upward trend was evident in both females and males. In females. The rates were rising by 10.26% (from 7.8%, 2528 in 2003 to 8.6%, 4458 in 2024) for a diet high in red meat and 9.62% (from 5.2%, 1691 in 2003 to 5.7%, 2991 in 2024) for a diet high in processed meat. In males, the rates were rising by 8.86% (from 7.9%, 3562 in 2003 to 8.6%, 6489 in 2024) for diet high in red meat and 7.55% (from 5.3%, 2374 in 2003 to 5.7%, 4314 in 2024) for diet high in processed meat. The rate of diet low in nuts and seeds decreased from 2.7% to 2.1% between 2003 and 2024, rising from 866 to 1104 among females and from 1197 to 1587 among males. Diet high in sugar and sweetened beverages contributing to T2DM-DALY showed an 8.00% decrease (from 2.5%, 1114 in 2003 to 2.3%, 1772 in 2024) in males, as no significance change in females.

### Kidney and urinary diseases

The overall impact of a high-sodium diet on chronic kidney disease has slightly declined, with the attributable proportion dropping from 6.7% (2229) in 2003 to 6.6% (4285) in 2024, reflecting a 1.49% decrease. This decline was more pronounced among females, whose rate fell from 5.7% (910) to 5.1% (1563), with a 10.53% reduction. In contrast, the rate for males rose slightly from 7.6% (1318) to 7.9% (2721), marking a 3.95% increase.

### Dietary risk-attributable deaths from non-communicable chronic diseases in Australia from 2003 to 2024

The research examined 13 dietary risk factors related to NCDs from 2003 to 2024, comparing their impact on mortality in Australia from 2003 to 2024 (Table 3).

**Table 3:** Deaths attributable to dietary risk factors for non-communicable diseases in Australia from 2003 to 2024.

	Risk Factors	Sex	2003	Total Death (n)	2024	2003–2024
			Attributable Death percentage, % (n)		Attributable Death percentage, %	Percentage Change, %
**Cancer and other neoplasms**						
Bowel cancer						
	All dietary risks	Female	24.5% (596)	2436	25.7%	4.90%
		Male	24.7% (703)	2850	25.8%	4.45%
		Person	24.6% (1298)	5285	25.7%	4.47%
	Diet high in processed meat	Female	1.9% (47)	2436	2.2%	15.79%
		Male	2.0% (56)	2850	2.2%	10.00%
		Person	2.0% (104)	5285	2.2%	10.00%
	Diet high in red meat	Female	5.0% (123)	2436	5.6%	12.00%
		Male	5.1% (146)	2850	5.7%	11.76%
		Person	5.1% (269)	5285	5.7%	11.76%
	Diet low in milk	Female	4.4% (107)	2436	4.6%	4.55%
		Male	4.4% (126)	2850	4.6%	4.55%
		Person	4.4% (233)	5285	4.6%	4.55%
	Diet low in whole grains & high fibre cereals	Female	15.2% (369)	2436	15.5%	1.97%
		Male	15.2% (434)	2850	15.6%	2.63%
		Person	15.2 (804)	5285	15.6%	
Breast cancer						
	All dietary risks	Female	3.0% (84)	2813	3.2%	6.67%
		Male	2.9% (0)	10	3.2%	10.34%
		Person	3.0% (84)	2823	3.2%	6.67%
	Diet high in red meat	Female	3.0% (84)	2813	3.2%	6.67%
		Male	2.9% (0)	10	3.2%	10.34%
		Person	3.0% (84)	2823	3.2%	6.67%
Lung cancer						
	All dietary risks	Female	3.7% (97)	2637	3.6%	−2.70%
		Male	3.6% (172)	4719	3.6%	0
		Person	3.7% (269)	7356	3.6%	−2.70%
	Diet low in fruit	Female	3.7% (97)	2637	3.6%	−2.70%
		Male	3.6% (172)	4719	3.6%	0
		Person	3.7% (269)	7356	3.6%	−2.70%
Oesophageal cancer						
	All dietary risks	Female	21.5% (81)	378	22.3%	3.72%
		Male	21.7% (171)	786	22.5%	4.78%
		Person	21.6% (252)	1164	22.4%	3.70%
	Diet low in fruit	Female	15.5% (58)	378	15.5%	0
		Male	15.9% (125)	786	15.8%	−0.63%
		Person	15.7% (183)	1164	15.7%	0
	Diet low in vegetables	Female	7.1% (27)	378	8.0%	12.68%
		Male	6.9% (55)	786	7.9%	14.49%
		Person	7.0% (81)	1164	7.9%	12.86%
**Cardiovascular diseases**						
Aortic aneurysm						
	All dietary	Female	4.6% (24)	527	3.9%	−15.22%
		Male	6.3% (52)	823	6.3%	0
		Person	5.6% (76)	1350	5.3%	−5.36%
	Diet high in sodium	Female	4.6% (24)	527	3.9%	−15.22%
		Male	6.3% (52)	823	6.3%	0
		Person	5.6% (76)	1350	5.3%	−5.36%
Atrial fibrillation and flutter						
	All dietary risks	Female	4.6% (28)	612	3.6%	−21.74%
		Male	5.9% (20)	332	5.9%	0
		Person	5.0% (48)	943	4.5%	−10.00%
	Diet high in sodium	Female	4.6% (28)	612	3.6%	−21.74%
		Male	5.9% (20)	332	5.9%	0
		Person	5.0% (48)	943	4.5%	−10.00%
Cardiomyopathy						
	All dietary risks	Female	4.7% (13)	276	4.2%	−10.64%
		Male	7.0% (41)	585	6.9%	−1.43%
		Person	6.3% (54)	861	6.0%	−4.76%
	Diet high in sodium	Female	4.7% (13)	276	4.2%	−10.64%
		Male	7.0% (41)	585	6.9%	−1.43%
		Person	6.3% (54)	861	6.0%	−4.76%
Coronary heart disease						
	All dietary risks	Female	45.9% (6183)	13,483	44.3%	−3.49%
		Male	49.5% (7278)	14,702	47.8%	−3.43%
		Person	47.8% (13,461)	28,185	46.4%	−2.93%
	Diet high in processed meat	Female	1.6% (217)	13,483	1.9%	18.75%
		Male	1.9% (276)	14,702	2.1%	10.53%
		Person	1.8% (494)	28,185	2.0%	11.11%
	Diet high in red meat	Female	3.9% (519)	13,483	4.5%	15.38%
		Male	4.4% (648)	14,702	4.8%	9.09%
		Person	4.1% (1167)	28,185	4.7%	14.63%
	Diet high in sodium	Female	5.8% (779)	13,483	4.7%	18.97%
		Male	7.8% (1152)	14,702	8.0%	2.56%
		Person	6.9% (1932)	28,185	6.7%	2.90%
	Diet high in sugar-sweetened beverages	Female	0.4% (55)	13,483	0.4%	0
		Male	0.8% (120)	14,702	0.8%	0
		Person	0.6% (175)	28,185	0.6%	0
	Diet low in fish & seafood	Female	1.7% (234)	13,483	1.4%	−17.65%
		Male	2.0% (288)	14,702	1.5%	−25.00%
		Person	1.9% (522)	28,185	1.5%	−21.05%
	Diet low in fruit	Female	4.3% (581)	13,483	4.3%	0
		Male	4.7% (689)	14,702	4.5%	−4.26%
		Person	4.5% (1270)	28,185	4.4%	−2.22%
	Diet low in legumes	Female	17.4% (2344)	13,483	16.4%	−5.75%
		Male	18.8% (2759)	14,702	17.3%	−7.98%
		Person	18.1% (5103)	28,185	16.9%	−6.63%
	Diet low in nuts & seeds	Female	11.3% (1521)	13,483	9.4%	−16.81%
		Male	12.0% (1771)	14,702	9.6%	−20.00%
		Person	11.7% (3293)	28,185	9.5%	−18.80%
	Diet low in polyunsaturated fats	Female	1.4% (195)	13,483	1.4%	0
		Male	1.7% (252)	14,702	1.7%	0
		Person	1.6% (447)	28,185	1.6%	0
	Diet low in vegetables	Female	4.9% (660)	13,483	5.5%	12.24%
		Male	5.1% (747)	14,702	5.7%	11.76%
		Person	5.0% (1407)	28,185	5.7%	14.00%
	Diet low in whole grains & high fibre cereals	Female	5.8% (783)	13,483	6.0%	3.45%
		Male	6.4% (941)	14,702	6.5%	1.56%
		Person	6.1% (1724)	28,185	6.3%	3.28%
Hypertensive heart disease						
	All dietary risks	Female	5.6% (27)	477	4.6%	−17.86%
		Male	8.0% (17)	218	8.1%	1.25%
		Person	6.4% (44)	695	6.0%	−6.25%
	Diet high in sodium	Female	5.6% (27)	477	4.6%	−17.86%
		Male	8.0% (17)	218	8.1%	1.25%
		Person	6.4% (44)	695	6.0%	−6.25%
Inflammatory heart disease						
	All dietary risks	Female	1.0% (1)	60	0.9%	−10.00%
		Male	1.3% (1)	77	1.4%	7.69%
		Person	1.2% (2)	137	1.2%	0
	Diet high in sodium	Female	1.0% (1)	60	0.9%	−10.00%
		Male	1.3% (1)	77	1.4%	7.69%
		Person	1.2% (2)	137	1.2%	0
Non-rheumatic valvular disease						
	All dietary risks	Female	3.7% (25)	685	3.0%	−18.92%
		Male	4.7% (26)	545	4.8%	2.13%
		Person	4.1% (51)	1230	3.8%	−7.32%
	Diet high in sodium	Female	3.7% (25)	685	3.0%	−18.92%
		Male	4.7% (26)	545	4.8%	2.13%
		Person	4.1% (51)	1230	3.8%	−7.32%
Peripheral vascular disease						
	All dietary risks	Female	3.8% (18)	479	3.0%	−21.05%
		Male	4.9% (18)	368	5.0%	2.04%
		Person	4.3% (36)	847	4.0%	−6.98%
	Diet high in sodium	Female	3.8% (18)	479	3.0%	−21.05%
		Male	4.9% (18)	368	5.0%	2.04%
		Person	4.3% (36)	847	4.0%	−6.98%
Rheumatic heart disease						
	All dietary risks	Female	4.0% (9)	213	3.4%	−15.00%
		Male	5.4% (5)	99	5.2%	−3.70%
		Person	4.4% (14)	312	4.1%	−6.82%
	Diet high in sodium	Female	4.0% (9)	213	3.4%	−15.00%
		Male	5.4% (5)	99	5.2%	−3.70%
		Person	4.4% (14)	312	4.1%	−6.82%
Stroke						
	All dietary risks	Female	20.1% (1550)	7710	20.1%	0
		Male	22.4% (1139)	5088	23.1%	3.13%
		Person	21.0% (2689)	12,798	21.4%	1.90%
	Diet high in red meat	Female	5.2% (398)	7710	5.8%	11.54%
		Male	5.7% (290)	5088	6.1%	7.02%
		Person	5.4% (688)	12,798	5.9%	9.26%
	Diet high in sodium	Female	5.5% (426)	7710	4.5%	−18.18%
		Male	7.2% (364)	5088	7.4%	2.78%
		Person	6.2% (790)	12,798	5.8%	−6.45%
	Diet low in fruit	Female	3.9% (297)	7710	3.8%	−2.56%
		Male	4.2% (212)	5088	4.0%	−4.76%
		Person	4.0% (509)	12,798	3.9%	−2.50%
	Diet low in vegetables	Female	5.5% (421)	7710	5.8%	5.45%
		Male	5.6% (287)	5088	5.9%	5.36%
		Person	5.5% (708)	12,798	5.9%	7.27%
	Diet low in whole grains & high fibre cereals	Female	2.0% (155)	7710	210.0%	5.00%
		Male	2.2% (113)	5088	2.2%	0
		Person	2.1% (269)	12,798	2.1%	0
**Endocrine disorders**						
Type 2 diabetes mellitus						
	All dietary risks	Female	23.8% (319)	1339	25.5%	7.14%
		Male	24.8% (373)	1502	25.7%	3.63%
		Person	24.4% (692)	2840	25.4%	4.10%
	Diet high in processed meat	Female	4.9% (66)	1339	5.8%	18.37%
		Male	5.0% (75)	1502	5.7%	14.00%
		Person	5.0% (141)	2840	5.8%	16.00%
	Diet high in red meat	Female	7.4% (99)	1339	8.3%	12.16%
		Male	7.6% (114)	1502	8.3%	9.21%
		Person	7.5% (213)	2840	8.3%	10.67%
	Diet high in sugar-sweetened beverages	Female	1.2% (17)	1339	1.2%	0
		Male	2.1% (32)	1502	2.1%	0
		Person	1.7% (48)	2840	1.7%	0
	Diet low in fruit	Female	6.3% (84)	1339	6.2%	−1.59%
		Male	6.3% (95)	1502	6.3%	0
		Person	6.3% (179)	2840	6.3%	0
	Diet low in nuts & seeds	Female	2.7% (37)	1339	2.3%	−14.81%
		Male	2.7% (41)	1502	2.3%	−14.81%
		Person	2.7% (78)	2840	2.3%	−14.81%
	Diet low in whole grains & high fibre cereals	Female	4.0% (53)	1339	4.1%	2.50%
		Male	4.0% (61)	1502	4.2%	5.00%
		Person	4.0% (114)	2840	4.1%	2.50%
**Kidney and urinary diseases**						
Chronic kidney disease						
	All dietary risks	Female	5.7% (62)	1085	4.8%	−15.79%
		Male	7.2% (72)	999	7.6%	5.56%
		Person	6.4% (134)	2085	6.2%	−3.13%
	Diet high in sodium	Female	5.7% (62)	1085	4.8%	−15.79%
		Male	7.2% (72)	999	7.6%	5.56%
		Person	6.4% (134)	2085	6.2%	−3.13%

N refers to the total number of deaths; % represents the proportion of total deaths attributable to each dietary risk factor.

### Cancer and other neoplasms

From 2003 to 2024, the mortality attributable to dietary risk factors across various cancers has generally increased. For bowel cancer, the most significant dietary risk is a diet high in processed meat, which rose from 1.9% (47) in 2003 to 2.2% in 2024, with a percentage change of 15.79% in females; the most significant dietary risk is diet high in red meat, which rose from 5.1% (146) in 2003 to 5.7% in 2024, with a percentage change of 11.76% in males. For breast cancer, the increase among males is notable, from 2.9% (0) in 2003 to 3.2% in 2024, with a 10.34% rise. In the case of lung cancer, diet low in fruit held steady at 3.6% both in 2003 (172) and in 2024 among males, while it showed a slight decline among females, from 3.7% (97) in 2003 to 3.6% in 2024, with a 2.70% decrease. The low vegetable intake for oesophageal cancer showed the most significant rise, increasing from 7.0% (81) in 2003 to 7.9% in 2024, with a 12.86% increase. The rate was slightly higher in males than in females. For males, it increased from 6.9% (55) in 2003 to 7.9% in 2024, representing a 14.49% increase; for females, it rose from 7.1% (27) to 8.0%, a percentage change of 12.68%.

### Cardiovascular diseases

As the only cardiovascular diseases showed an upward trend among persons, stroke saw an increase in all dietary risks attributable mortality, rising by 1.90%, from 21.0% (2689) in 2003 to 21.4% in 2024, which was mainly driven by diet high in red meat (from 5.4%, 688 in 2003 to 5.9% in 2024, percentage change 9.26%) and diet low in vegetables (from 5.5%, 708 in 2003 to 5.9% in 2024, percentage change 7.27%). The diet high in sodium attributed to AF/AFL experienced the largest decline, with the overall diet-attributable mortality rate decreasing by 10.00%, from 5.0% (48) in 2003 to 4.5% in 2024. In females, the largest increase was a diet high in sodium in CHD, with an 18.97% rise (from 5.8%, 779 in 2003 to 4.7% in 2024). The largest decrease in diet high in sodium in AF/AFL, with a 21.74% percentage change decrease (from 4.6%, 28 in 2003 to 3.6% in 2024). In males, the largest increase was a diet low in vegetables, which was contributing to mortality observed in CHD, with an 11.76% rise (from 5.1%, 747 in 2003 to 5.7% in 2024). The largest decrease was diet low in fish and seafood, contributing to mortality observed in CHD with a 25.00% decrease (from 2.0%, 288 in 2003 to 1.5% in 2024).

### Endocrine disorders

Mortality attributable to dietary risks related to T2DM has shown an overall upward trend. The total attributable proportion increased from 24.4% (692) in 2003 to 25.4% in 2024, representing a 4.10% percentage change. The increase was more pronounced among females at 7.14%, rising from 23.8% (319) in 2003 to 25.5% in 2024. In comparison, males showed a 3.63% increase, from 24.8% (373) to 25.7% over the same period. Among specific dietary factors, a diet high in processed meat showed a total increase of 16.00%, the most significant increase in attributable mortality, rising from 5.0% (141) in 2003 to 5.8% in 2024. The rise was more important in females, increasing from 4.9% (66) in 2003 to 5.8% in 2024, representing an 18.37% rise. Compared to males, which got the increase from 5.0% (75) to 5.7%, a 14.00% rise. In contrast, the attributable mortality from a diet low in nuts and seeds decreased significantly, dropping from 2.7% (78) in 2003 to 2.3% in 2024, representing a total reduction of 14.81%. This trend was consistent across both males and females.

### Kidney and urinary diseases

From 2003 to 2024, the diet-related mortality attributable to a diet high in sodium in CKD showed a slight decline among persons, decreasing from 6.4% (134) in 2003 to 6.2% in 2024, with a reduction percentage change of 3.13%. A clear sex difference existed: among females, the rate dropped from 5.7% (62) in 2003 to 4.8% in 2024, a 15.79% decrease; whereas in males, it rose from 7.2% (72) in 2003 to 7.6% in 2024, marking a 5.56% increase.

## Discussion

Previous research using GBD data has offered valuable cross-country insights into the global effects of dietary risks.^14^ In 2021, across all SDI countries, the primary dietary risk factors for cardiovascular mortality and DALYs remained unchanged compared to 2019, despite a decrease in their prevalence.^15^^,^^16^ Excessive red meat consumption became the dietary factor most strongly associated with global cancer deaths and DALYs, although its prevalence declined in high-SDI countries.^17^ By using AIHW data, this study complements GBD findings that reflect the local healthcare context of Australia, which highlights the complexity and heterogeneity of changes in the dietary risk factors attributable to the burden of NCDs. Although the ASRs of many NCDs declined between 2003 and 2024, the number and proportion of disease burdens attributable to dietary risks exhibited diverse and complex trends. Compared with the GBD study, our analysis reached highly consistent conclusions regarding high sodium intake and the increased burden of CHD.^18^ Moreover, a recent large prospective study reported that low vegetable intake was significantly associated with ischemic stroke risk,^19^ which echoes our finding—in a female-stratified analysis—that low vegetable intake increased the burden of oesophageal cancer.^20^^,^^21^

Among cancers, red meat was the most significant dietary factor contributing to increased disease burden for both bowel and breast cancers, with a more substantial impact observed in males compared to females. Low vegetable intake notably increased the burden disease percentage for oesophageal cancer, while the effect of low fruit intake appeared to be well controlled in both lung and oesophageal cancers. For CVDs, inflammatory heart disease DALYs attributable to high sodium intake showed a marked increase across the total population, especially among males. In females, a significant increase was observed in CHD-related DALYs attributable to processed meat consumption. The stroke-related disease burden increased due to both high red meat intake and low vegetable intake. Conversely, DALYs for AF/AFL attributable to high sodium intake significantly declined across the population. Another contributing factor with a declining impact was low fish and seafood intake, which reduced inflammatory heart disease DALYs in males and CHD-related DALYs in females—indicating effective control of these dietary risks. In females, CHD-related mortality attributable to high processed meat intake increased significantly, while AF/AFL mortality attributable to high sodium intake significantly decreased. In males, CHD mortality attributable to low vegetable intake increased, while CHD mortality due to low fish and seafood intake decreased notably. In diabetes, regardless of sex, high processed meat intake was the leading contributor to increased mortality, while high red meat intake was the main driver of increased DALYs. Conversely, low intake of nuts and seeds was the most significant factor associated with reducing disease burden. Although the overall DALYs for CKD declined, CKD-related burden attributable to high sodium intake increased among males.

In Australia, high consumption of processed meat is closely associated with an increased burden of CHD, T2DM, and bowel cancer. The findings of the present study are consistent with previous studies that have indicated a correlation between high processed meat consumption and an increased risk of CHD, hypertension, stroke, and T2DM.^12^^,^^22^ This may be linked to certain preservatives found in processed meat, such as nitrates, which have been associated with endothelial dysfunction, atherosclerosis, and insulin resistance.^23^^,^^24^

The GBD study indicates that, compared to 1990, both the number of deaths and DALY caused by CHD due to a diet high in sodium intake significantly increased in 2021.^25^ In Australia, excessive dietary salt intake is closely linked to a higher burden of CHD, hypertension, and T2DM. Studies have confirmed a positive correlation between high sodium intake and CVDs, likely due to the effects of a diet high in sodium on blood pressure, kidney function, and metabolic syndrome.^26^ A study on hypertensive patients found that those consuming a high-salt diet had higher blood pressure levels than those on a low-salt diet, increasing their risk of CVDs.^27^ Additionally, a diet high in sodium is closely associated with insulin resistance,^28^ a significant risk factor for T2DM. Research also shows a positive correlation between high salt consumption and the incidence of obesity and hypertension.^29^ Excessive sodium intake may impair vascular function, contribute to arterial stiffness, and elevate the risk of CVDs.^30^ Recent studies have highlighted the impact of high sodium intake on kidney function, revealing that it can raise blood pressure in CKD and accelerate cardiovascular complications.^31^ Excessive sodium in certain processed foods may further exacerbate atherosclerosis by inducing vasoconstriction and increasing oxidative stress.^32^

A large-scale prospective study found that individuals with low vegetable intake had a significantly higher risk of ischemic stroke and CVDs compared to those with adequate vegetable consumption.^33^ Low vegetable intake may lead to endothelial dysfunction, increased inflammatory responses, elevated blood pressure, and abnormal lipid levels, thereby raising the risk of cardiovascular and cerebrovascular events.^34^ Additionally, dietary fiber, antioxidants, and phytosterols in vegetables play a crucial role in vascular health, and a deficiency in these nutrients may accelerate the progression of CHD.^35^ Antioxidants and polyphenolic compounds in vegetables help reduce inflammation levels. In contrast, insufficient vegetable intake may increase inflammatory markers, such as C-reactive protein, thereby heightening the risk of vascular damage.^36^ Moreover, potassium in vegetables is essential for maintaining blood pressure stability, and low potassium intake has been linked to an increased risk of hypertension and stroke.^37^ Furthermore, inadequate vegetable intake may increase vascular resistance and reduce arterial elasticity, elevating the risk of ischemic stroke, as evidenced by a large-scale study in European populations that found individuals with lower vegetable intake had a significantly higher risk of stroke, particularly ischemic stroke, compared to those with higher vegetable consumption.^38^

This study has shown significant differences in the impact of dietary risk factors on NCDs between sex. Men are more affected by processed meat consumption about CHD, T2DM, and bowel cancer. In contrast, women exhibit higher sensitivity to the relationship between high sodium intake and CHD. Additionally, inadequate whole grain intake has a more significant impact on bowel cancer risk in men, further emphasizing the necessity of personalized dietary guidance based on sex. A systematic review found that high consumption of red and processed meat is significantly associated with an increased risk of bowel cancer in men. In contrast, females avoid high-fat foods and consume more milk, fruits, vegetables, and dietary fiber. As a result, women exhibit lower sensitivity to this dietary factor.^39^^,^^40^ Additionally, the study indicated that lower whole grain intake is linked to an increased risk of bowel cancer in men, while the associated risk for women is comparatively lower.^41^ In terms of CVDs, a study on dietary factors and heart disease incidence found that high sodium intake has a particularly significant impact on hypertension in women.^42^ Similarly, the study indicated that women are more susceptible than men to the effects of high salt intake in their diet, leading to elevated blood pressure and an increased risk of hypertension-related heart disease,^43^ which is related to the notion that testosterone represses salt appetite hormones and chromosomes influence dietary salt intake.^44^ These studies emphasize the importance of considering sex factors when formulating public health policies and nutritional guidelines to more precisely prevent and manage diet-related NCDs.

New evidence on how specific dietary risk factors are differentially associated with chronic disease burdens by sex and disease type in the Australian population from 2003 to 2024. DALYs and mortality attributable to red meat and processed meat continue to increase in diseases like breast cancer, T2DM, and CHD. Clinicians should integrate specific food risk assessments into routine evaluations and strengthen dietary interventions, particularly for high-risk patients. The study supports modifying dietary guidance and risk communication protocols to prioritize reduction in sodium and processed meat intake. This can be incorporated into national dietary recommendations and chronic disease management frameworks. The sex- and disease-specific dietary trends revealed in this study offer clinically actionable insights. These findings support a more personalized, risk-based dietary counseling approach and refinement of existing clinical and public health guidelines.

Although the overall burden of NCDs attributable to dietary risks in Australia declined from 2003 to 2024, the diet-related burden of diseases such as breast cancer, inflammatory heart disease, and T2DM increased. Clear sex differences were also observed: men were more affected by red and processed meat intake in conditions like T2DM and bowel cancer, while women were more susceptible to adverse cardiovascular outcomes associated with high sodium intake. These findings highlight the need to strengthen sex-specific dietary guidance and risk assessment in clinical practice. Future studies should explore how diet interacts with other lifestyle and environmental factors to shape NCD outcomes. Integrating dietary data into risk stratification models could enhance early intervention strategies for CHD, T2DM, and cancer. The study highlights modifiable dietary risks as ongoing, clinically actionable contributors to NCD burden in Australia. Our findings support personalized nutrition counseling, improved dietary screening protocols, and a future research agenda that integrates dietary data into predictive, sex-responsive clinical frameworks.

Several limitations existed. First, the counts of death on 2024 were missed, which led accurate analysis could not be made. Second, although we analyzed 13 majors dietary risk factors, data constraints prevented us from including other important variables such as overall dietary patterns, food processing methods, or nutrient details, such as protein, fat, carbohydrate, mineral. Third, we did not adjust for lifestyle or socioeconomic factors—such as physical activity, alcohol consumption, income, and education level—which may confound the relationship between dietary risks and health outcomes.

Future research should aim to incorporate longitudinal, individual-level dietary intake data linked with clinical outcomes to establish stronger causal inferences between specific dietary risks and chronic disease progression. In particular, prospective cohort studies or randomized controlled trials focused on high-risk food categories—such as red and processed meats or sodium-rich diets—are needed to validate intervention strategies. Additionally, integrating socioeconomic and behavioral factors—such as affordability, cultural food preferences, and health literacy—could enhance the development of targeted dietary guidelines and public health campaigns. Finally, expanding comparative studies to other countries would help assess the global relevance of dietary risks identified in the Australian context.

From 2003 to 2024, the dietary risk–attributable burden of NCDs in Australia declined overall, but increased in specific conditions such as breast cancer, T2DM, and inflammatory heart disease. These changes were driven mainly by high intake of sodium, red meat, and processed meat. The analysis also revealed clear sex differences: women were more affected by sodium-related CHD, while men showed higher burdens from red and processed meats. These conclusions are directly supported by national DALY and mortality data.

## Contributors

YX Xu contributed to the original draft writing, data visualization and reviewed and edited the manuscripts. J. Sun conceptualized the study, analyzed the data, and reviewed and edited the manuscripts. All authors have verified the underlying data, read and approved the final version of the manuscript for submission.

## Data sharing statement

Data in this study were obtained from the Australian Institute of Health and Welfare, Australian Burden of Disease Study 2024. All aggregated data are publicly available at: https://www.aihw.gov.au. No additional access restrictions apply.

## Declaration of interests

No competing interests were declared by all authors.

## Appendix A Supplementary data

Supplementary 2nd Revision
