# Supplementary material for: Dietary attribution to burden of chronic disease in Australia: a systematic analysis of the Australian Institute of health and welfare 2024 national burden of disease dataset
Source: eClinicalMedicine. 2025 Aug 11;87:103418. doi: 10.1016/j.eclinm.2025.103418 (PMC12359163; doi:10.1016/j.eclinm.2025.103418)
Supplement: Supplementary 2nd Revision [file mmc1.docx]

| Disease Group | Disease | Sex | 2003 ASR (95% UI) | 2011 ASR (95% UI) | 2015 ASR (95% UI) | 2018 ASR (95% UI) | 2024 ASR (95% UI) | 2003-2024 APC (95% CI） | P |
| --- | --- | --- | --- | --- | --- | --- | --- | --- | --- |
| **Cancer and other neoplasms** |  |  |  |  |  |  |  |  |  |
|  | Bowel cancer | Female | 2.951 (2.922, 2.980) | 2.313 (2.289, 2.336) | 2.326 (2.303, 2.349) | 2.160 (2.139, 2.182) | 1.824 (1.805, 1.842) | -2.130% (-2.722%, -1.541%) | < 0.001 |
|  |  | Male | 4.359 (4.323, 4.396) | 3.468 (3.438, 3.497) | 3.271 (3.244, 3.299) | 2.994 (2.968, 3.020) | 2.506 (2.484, 2.528) | -2.537% (-2.981%, -2.085%) | < 0.001 |
|  |  | Person | 3.624 (3.600, 3.647) | 2.871 (2.852, 2.890) | 2.78 (2.762, 2.798) | 2.561 (2.545, 2.578) | 2.151 (2.136, 2.165) | -2.360% (-2.975%, -1.719%) | < 0.001 |
|  | Breast cancer | Female | 5.458 (5.417, 5.499) | 4.621 (4.587, 4.656) | 4.117 (4.085, 4.148) | 3.903 (3.873, 3.933) | 3.514 (3.487, 3.542) | -2.120% (-2.401%, -1.836%) | < 0.001 |
|  |  | Male | 0.023 (0.020, 0.025) | 0.025 (0.022, 0.027) | 0.030 (0.027, 0.032) | 0.040 (0.037, 0.043) | 0.029 (0.027, 0.032) | 1.744% (-1.151%, 4.987%) | 0.207 |
|  |  | Person | 2.796 (2.775, 2.817) | 2.372 (2.354, 2.389) | 2.124 (2.108, 2.140) | 2.023 (2.007, 2.038) | 1.822 (1.808, 1.836) | -2.056% (-2.269%, -1.845%) | < 0.001 |
|  | Lung cancer | Female | 3.575 (3.543, 3.608) | 3.632 (3.603, 3.662) | 3.595 (3.567, 3.623) | 3.358 (3.332, 3.384) | 2.917 (2.894, 2.940) | -0.983% (-2.201%, 0.366%) | 0.144 |
|  |  | Male | 6.940 (6.893, 6.988) | 5.919 (5.881, 5.958) | 5.193 (5.159, 5.227) | 4.803 (4.771, 4.834) | 3.922 (3.895, 3.949) | -2.686% (-3.426%, -1.905%) | < 0.001 |
|  |  | Person | 5.171 (5.143, 5.200) | 4.722 (4.698, 4.746) | 4.355 (4.333, 4.377) | 4.049 (4.028, 4.069) | 3.394 (3.376, 3.412) | -1.984% (-2.439%, -1.520%) | < 0.001 |
|  | Oesophageal cancer | Female | 0.394 (0.384, 0.404) | 0.302 (0.294, 0.311) | 0.301 (0.293, 0.309) | 0.232 (0.226, 0.239) | 0.234 (0.228, 0.241) | -2.569% (-3.828%, -1.306%) | < 0.001 |
|  |  | Male | 1.276 (1.256, 1.296) | 1.174 (1.157, 1.191) | 1.064 (1.048, 1.080) | 1.039 (1.024, 1.054) | 0.978 (0.964, 0.992) | -1.311% (-1.708%, -0.892%) | < 0.001 |
|  |  | Person | 0.824 (0.813, 0.835) | 0.727 (0.718, 0.737) | 0.672 (0.663, 0.681) | 0.624 (0.616, 0.632) | 0.594 (0.587, 0.602) | -1.608% (-1.874%, -1.333%) | < 0.001 |
| **Cardiovascular diseases** |  |  |  |  |  |  |  |  |  |
|  | Aortic aneurysm | Female | 0.363 (0.353, 0.372) | 0.237 (0.230, 0.244) | 0.225 (0.219, 0.232) | 0.193 (0.188, 0.199) | 0.194 (0.188, 0.199) | -3.048% (-5.579%, -0.426%) | 0.018 |
|  |  | Male | 0.911 (0.894, 0.927) | 0.649 (0.636, 0.662) | 0.499 (0.488, 0.510) | 0.502 (0.492, 0.513) | 0.450 (0.441, 0.460) | -3.472% (-5.469%, -1.523%) | < 0.001 |
|  |  | Person | 0.617 (0.608, 0.626) | 0.435 (0.428, 0.442) | 0.357 (0.351, 0.364) | 0.343 (0.337, 0.349) | 0.318 (0.312, 0.323) | -3.269% (-5.381%, -1.162%) | < 0.001 |
|  | Atrial fibrillation and flutter | Female | 0.758 (0.745, 0.771) | 0.821 (0.809, 0.834) | 0.940 (0.927, 0.952) | 0.950 (0.938, 0.962) | 0.950 (0.939, 0.961) | 1.117% (0.268%, 2.124%) | 0.006 |
|  |  | Male | 1.162 (1.143, 1.180) | 1.259 (1.239, 1.279) | 1.387 (1.370, 1.404) | 1.473 (1.456, 1.489) | 1.482 (1.466, 1.497) | 1.225% (0.487%, 2.178%) | < 0.001 |
|  |  | Person | 0.954 (0.943, 0.965) | 1.036 (1.025, 1.048) | 1.160 (1.149, 1.170) | 1.204 (1.194, 1.214) | 1.207 (1.197, 1.217) | 1.170% (0.384%, 2.132%) | < 0.001 |
|  | Cardiomyopathy | Female | 0.594 (0.572, 0.617) | 0.503 (0.491, 0.515) | 0.588 (0.570, 0.606) | 0.639 (0.613, 0.666) | 0.467 (0.452, 0.482) | -0.648% (-4.477%, 3.119%) | 0.585 |
|  |  | Male | 1.725 (1.683, 1.766) | 1.259 (1.239, 1.279) | 1.268 (1.248, 1.288) | 1.439 (1.404, 1.474) | 1.127 (1.106, 1.148) | -1.520% (-4.749%, 1.744%) | 0.296 |
|  |  | Person | 1.145 (1.122, 1.169) | 0.865 (0.854, 0.877) | 0.916 (0.903, 0.929) | 1.034 (1.013, 1.056) | 0.788 (0.776, 0.801) | -1.251% (-3.199%, 0.675%) | 0.206 |
|  | Coronary heart disease | Female | 8.496 (8.451, 8.541) | 5.451 (5.417, 5.486) | 4.467 (4.438, 4.496) | 3.933 (3.907, 3.959) | 3.136 (3.114, 3.158) | -4.742% (-5.493%, -4.061%) | < 0.001 |
|  |  | Male | 20.403 (20.325, 20.481) | 14.133 (14.074,14.191) | 12.068 (12.016,12.121) | 10.85 (10.802,10.898) | 9.359 (9.317, 9.401) | -3.740% (-4.281%, -3.250%) | < 0.001 |
|  |  | Person | 14.166 (14.122, 14.210) | 9.62 (9.586, 9.653) | 8.129 (8.100, 8.159) | 7.262 (7.235, 7.289) | 6.123 (6.100, 6.147) | -4.016% (-4.578%, -3.517%) | < 0.001 |
|  | Hypertensive heart disease | Female | 0.207 (0.200, 0.214) | 0.183 (0.177, 0.189) | 0.233 (0.227, 0.239) | 0.216 (0.210, 0.222) | 0.205 (0.200, 0.211) | 0.181% (-1.298%, 1.943%) | 0.715 |
|  |  | Male | 0.259 (0.250, 0.268) | 0.243 (0.235, 0.251) | 0.357 (0.348, 0.367) | 0.323 (0.315, 0.332) | 0.392 (0.383, 0.400) | 2.252% (0.434%, 4.650%) | 0.010 |
|  |  | Person | 0.238 (0.233, 0.244) | 0.216 (0.211, 0.221) | 0.298 (0.292, 0.303) | 0.272 (0.267, 0.277) | 0.298 (0.293, 0.303) | 1.302% (-0.072%, 2.992%) | 0.072 |
|  | Inflammatory heart disease | Female | 0.263 (0.243, 0.282) | 0.165 (0.158, 0.173) | 0.162 (0.150, 0.173) | 0.177 (0.170, 0.184) | 0.176 (0.169, 0.183) | -0.899% (-3.575%, 2.437%) | 0.615 |
|  |  | Male | 0.202 (0.194, 0.211) | 0.249 (0.240, 0.258) | 0.207 (0.199, 0.215) | 0.374 (0.362, 0.387) | 0.312 (0.303, 0.321) | 2.373% (-0.178%, 5.301%) | 0.072 |
|  |  | Person | 0.210 (0.203, 0.218) | 0.205 (0.200, 0.211) | 0.168 (0.163, 0.173) | 0.264 (0.258, 0.271) | 0.242 (0.237, 0.248) | 1.119% (-1.136%, 4.015%) | 0.346 |
|  | Non-rheumatic valvular disease | Female | 0.666 (0.653, 0.679) | 0.634 (0.623, 0.645) | 0.559 (0.549, 0.569) | 0.559 (0.510, 0.529) | 0.503 (0.495, 0.512) | -1.481% (-2.528%, -0.336%) | 0.006 |
|  |  | Male | 0.924 (0.907, 0.940) | 0.927 (0.911, 0.944) | 0.829 (0.815, 0.842) | 0.829 (0.712, 0.735) | 0.682 (0.672, 0.693) | -1.636% (-3.072%, 0.006%) | 0.051 |
|  |  | Person | 0.784 (0.774, 0.795) | 0.771 (0.761, 0.781) | 0.686 (0.678, 0.695) | 0.686 (0.608, 0.623) | 0.588 (0.581, 0.594) | -1.548% (-2.742%, -0.213%) | 0.016 |
|  | Peripheral vascular disease | Female | 0.268 (0.260, 0.276) | 0.174 (0.168, 0.180) | 0.157 (0.151, 0.162) | 0.157 (0.164, 0.175) | 0.145 (0.140, 0.150) | -2.823% (-3.999%, -1.696%) | < 0.001 |
|  |  | Male | 0.467 (0.450, 0.483) | 0.303 (0.294, 0.311) | 0.263 (0.256, 0.270) | 0.263 (0.269, 0.284) | 0.235 (0.229, 0.241) | -2.881% (-4.624%, -0.900%) | 0.001 |
|  |  | Person | 0.361 (0.352, 0.370) | 0.234 (0.229, 0.239) | 0.207 (0.202, 0.212) | 0.207 (0.215, 0.225) | 0.187 (0.183, 0.191) | -2.795% (-4.337%, -1.085%) | < 0.001 |
|  | Rheumatic heart disease (including acute rheumatic fever) | Female | 0.263 (0.254, 0.272) | 0.232 (0.224, 0.240) | 0.206 (0.199, 0.213) | 0.206 (0.174, 0.187) | 0.149 (0.144, 0.155) | -2.684% (-3.900%, -1.437%) | < 0.001 |
|  |  | Male | 0.165 (0.157, 0.172) | 0.135 (0.129, 0.141) | 0.150 (0.144, 0.156) | 0.105 (0.130, 0.142) | 0.106 (0.102, 0.111) | -1.763% (-3.994%, 0.621%) | 0.152 |
|  |  | Person | 0.216 (0.210, 0.222) | 0.185 (0.180, 0.190) | 0.179 (0.175, 0.184) | 0.179 (0.155, 0.164) | 0.129 (0.125, 0.132) | -2.327% (-3.445%, -1.162%) | < 0.001 |
|  | Stroke | Female | 4.314 (4.280, 4.348) | 3.138 (3.111, 3.166) | 2.563 (2.541, 2.586) | 2.563 (2.502, 2.551) | 2.020 (2.001, 2.040) | -3.573% (-4.167%, -3.024%) | < 0.001 |
|  |  | Male | 5.360 (5.320, 5.400) | 3.838 (3.807, 3.869) | 3.179 (3.153, 3.205) | 3.179 (3.082, 3.134) | 2.593 (2.571, 2.615) | -3.437% (-4.250%, -2.667%) | < 0.001 |
|  |  | Person | 4.795 (4.770, 4.821) | 3.479 (3.459, 3.500) | 2.862 (2.845, 2.879) | 2.862 (2.788, 2.822) | 2.294 (2.280, 2.309) | -3.485% (-4.067%, -2.947%) | < 0.001 |
| **Endocrine disorders** |  |  |  |  |  |  |  |  |  |
|  | Type 2 diabetes mellitus | Female | 2.203 (2.178, 2.229) | 2.434 (2.410, 2.458) | 2.354 (2.331, 2.377) | 2.354 (2.166, 2.208) | 2.081 (2.061, 2.100) | -0.452% (-2.288%, 1.625%) | 0.521 |
|  |  | Male | 3.508 (3.475, 3.540) | 3.459 (3.429, 3.488) | 3.433 (3.405, 3.461) | 3.433 (3.409, 3.463) | 3.373 (3.348, 3.398) | -0.178% (-0.220%, -0.135%) | < 0.001 |
|  |  | Person | 2.824 (2.804, 2.845) | 2.923 (2.905, 2.942) | 2.870 (2.852, 2.888) | 2.870 (2.776, 2.810) | 2.703 (2.687, 2.718) | -0.275% (-0.931%, 0.466%) | 0.346 |
| **Kidney and urinary diseases** |  |  |  |  |  |  |  |  |  |
|  | Chronic kidney disease | Female | 0.929 (0.914, 0.945) | 1.267 (1.240, 1.293) | 1.554 (1.521, 1.587) | 1.554 (1.090, 1.136) | 1.178 (1.158, 1.199) | 1.097% (-0.965%, 3.089%) | 0.327 |
|  |  | Male | 1.380 (1.358, 1.402) | 1.473 (1.444, 1.502) | 1.769 (1.744, 1.793) | 1.769 (1.377, 1.424) | 1.553 (1.529, 1.576) | 0.501% (-0.828%, 1.783%) | 0.345 |
|  |  | Person | 1.138 (1.125, 1.151) | 1.354 (1.334, 1.373) | 1.647 (1.626, 1.667) | 1.647 (1.233, 1.266) | 1.357 (1.341, 1.373) | 0.781% (-0.851%, 2.336%) | 0.333 |

**Supplementary Table S1.** ASRs of DALY for non-communicable diseases in Australia by disease and sex from 2003 to 2024.

Abbreviations: ASR, age-standardized rate per 1,000 population. DALY, disability-adjusted life year. APC, annual percentage change. CI, confidence interval. UI, uncertainty interval.

**ASR**


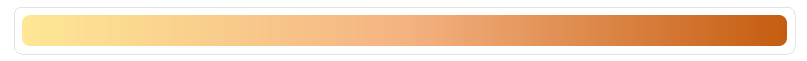
0.000 20.403

| Disease Group | Disease | Sex | 2003 ASR (95% UI) | 2011 ASR (95% UI) | 2015 ASR (95% UI) | 2018 ASR (95% UI) | 2024 ASR (95% UI) | 2003-2024 APC (95% CI） | P |
| --- | --- | --- | --- | --- | --- | --- | --- | --- | --- |
| **Cancer and other neoplasms** |  |  |  |  |  |  |  |  |  |
|  | Bowel cancer | Female | 2.805 (2.776, 2.833) | 2.175 (2.152, 2.197) | 2.197 (2.175, 2.220) | 2.036 (2.015, 2.057) | 1.710 (1.692, 1.728) | -2.184% (-2.793%, -1.588%) | < 0.001 |
|  |  | Male | 4.140 (4.105, 4.176) | 3.266 (3.238, 3.295) | 3.087 (3.060, 3.113) | 2.819 (2.794, 2.844) | 2.351 (2.329, 2.372) | -2.590% (-3.054%, -2.117%) | < 0.001 |
|  |  | Person | 3.444 (3.422, 3.467) | 2.704 (2.686, 2.722) | 2.626 (2.608, 2.643) | 2.413 (2.397, 2.429) | 2.018 (2.004, 2.032) | -2.414% (-3.062%, -1.741%) | < 0.001 |
|  | Breast cancer | Female | 4.963 (4.924, 5.002) | 4.119 (4.086, 4.152) | 3.612 (3.582, 3.641) | 3.411 (3.382, 3.439) | 3.023 (2.998, 3.049) | -2.384% (-2.693%, -2.077%) | < 0.001 |
|  |  | Male | 0.018 (0.016, 0.021) | 0.020 (0.018, 0.022) | 0.025 (0.022, 0.027) | 0.035 (0.032, 0.038) | 0.024 (0.022, 0.026) | 1.940% (-1.354%, 5.675%) | 0.215 |
|  |  | Person | 2.538 (2.518, 2.558) | 2.110 (2.094, 2.127) | 1.861 (1.846, 1.876) | 1.766 (1.751, 1.780) | 1.566 (1.552, 1.579) | -2.316% (-2.555%, -2.083%) | < 0.001 |
|  | Lung cancer | Female | 3.522 (3.490, 3.554) | 3.572 (3.543, 3.602) | 3.532 (3.504, 3.560) | 3.294 (3.269, 3.320) | 2.849 (2.826, 2.872) | -1.020% (-2.244%, 0.331%) | 0.134 |
|  |  | Male | 6.831 (6.784, 6.878) | 5.820 (5.783, 5.858) | 5.100 (5.067, 5.134) | 4.714 (4.683, 4.745) | 3.841 (3.814, 3.867) | -2.708% (-3.454%, -1.925%) | < 0.001 |
|  |  | Person | 5.094 (5.065, 5.122) | 4.645 (4.621, 4.669) | 4.279 (4.257, 4.301) | 3.974 (3.953, 3.994) | 3.320 (3.303, 3.338) | -2.014% (-2.475%, -1.544%) | < 0.001 |
|  | Oesophageal cancer | Female | 0.387 (0.376, 0.397) | 0.296 (0.288, 0.304) | 0.295 (0.287, 0.303) | 0.227 (0.221, 0.234) | 0.230 (0.223, 0.236) | -2.583% (-3.859%, -1.304%) | < 0.001 |
|  |  | Male | 1.256 (1.236, 1.275) | 1.154 (1.137, 1.171) | 1.045 (1.030, 1.061) | 1.020 (1.006, 1.035) | 0.960 (0.947, 0.974) | -1.323% (-1.718%, -0.906%) | < 0.001 |
|  |  | Person | 0.811 (0.800, 0.822) | 0.715 (0.705, 0.724) | 0.66 (0.651, 0.669) | 0.613 (0.605, 0.621) | 0.583 (0.576, 0.591) | -1.621% (-1.895%, -1.340%) | < 0.001 |
| **Cardiovascular diseases** |  |  |  |  |  |  |  |  |  |
|  | Aortic aneurysm | Female | 0.362 (0.352, 0.372) | 0.237 (0.230, 0.244) | 0.225 (0.218, 0.232) | 0.193 (0.188, 0.199) | 0.194 (0.188, 0.199) | -3.046% (-5.572%, -0.428%) | 0.018 |
|  |  | Male | 0.909 (0.893, 0.926) | 0.649 (0.636, 0.661) | 0.499 (0.488, 0.509) | 0.502 (0.492, 0.513) | 0.450 (0.441, 0.459) | -3.468% (-5.457%, -1.526%) | < 0.001 |
|  |  | Person | 0.617 (0.607, 0.626) | 0.435 (0.428, 0.442) | 0.357 (0.351, 0.363) | 0.343 (0.337, 0.349) | 0.317 (0.312, 0.323) | -3.266% (-5.374%, -1.162%) | < 0.001 |
|  | Atrial fibrillation and flutter | Female | 0.216 (0.210, 0.223) | 0.267 (0.261, 0.274) | 0.306 (0.299, 0.313) | 0.261 (0.255, 0.267) | 0.273 (0.268, 0.279) | 0.787% (-1.046%, 2.919%) | 0.365 |
|  |  | Male | 0.243 (0.234, 0.251) | 0.316 (0.302, 0.329) | 0.313 (0.305, 0.320) | 0.298 (0.291, 0.305) | 0.319 (0.311, 0.326) | 1.065% (-0.268%, 2.755%) | 0.114 |
|  |  | Person | 0.231 (0.226, 0.236) | 0.293 (0.286, 0.301) | 0.312 (0.307, 0.317) | 0.28 (0.275, 0.284) | 0.296 (0.291, 0.300) | 0.917% (-0.583%, 2.744%) | 0.206 |
|  | Cardiomyopathy | Female | 0.538 (0.515, 0.560) | 0.452 (0.440, 0.464) | 0.539 (0.522, 0.557) | 0.59 (0.564, 0.616) | 0.419 (0.405, 0.434) | -0.625% (-4.949%, 3.624%) | 0.635 |
|  |  | Male | 1.609 (1.568, 1.650) | 1.160 (1.140, 1.179) | 1.173 (1.154, 1.192) | 1.342 (1.308, 1.377) | 1.033 (1.013, 1.054) | -1.569% (-5.077%, 1.957%) | 0.303 |
|  |  | Person | 1.064 (1.041, 1.087) | 0.793 (0.782, 0.804) | 0.847 (0.834, 0.860) | 0.963 (0.942, 0.985) | 0.719 (0.707, 0.732) | -1.291% (-3.151%, 0.550%) | 0.178 |
|  | Coronary heart disease | Female | 6.791 (6.752, 6.831) | 4.154 (4.123, 4.184) | 3.402 (3.377, 3.427) | 2.936 (2.913, 2.958) | 2.335 (2.316, 2.355) | -5.100% (-5.947%, -4.357%) | < 0.001 |
|  |  | Male | 17.021 (16.949, 17.092) | 11.515 (11.462, 11.568) | 9.885 (9.837, 9.933) | 8.789 (8.745, 8.832) | 7.669 (7.630, 7.707) | -3.842% (-4.417%, -3.308%) | < 0.001 |
|  |  | Person | 11.683 (11.643, 11.723) | 7.700 (7.670, 7.730) | 6.534 (6.507, 6.560) | 5.759 (5.735, 5.784) | 4.900 (4.879, 4.921) | -4.180% (-4.820%, -3.614%) | < 0.001 |
|  | Hypertensive heart disease | Female | 0.203 (0.196, 0.210) | 0.180 (0.174, 0.185) | 0.230 (0.223, 0.236) | 0.213 (0.207, 0.218) | 0.202 (0.197, 0.207) | 0.200% (-1.300%, 1.982%) | 0.697 |
|  |  | Male | 0.255 (0.247, 0.264) | 0.240 (0.233, 0.248) | 0.355 (0.346, 0.364) | 0.321 (0.312, 0.329) | 0.389 (0.380, 0.398) | 2.286% (0.498%, 4.662%) | 0.007 |
|  |  | Person | 0.234 (0.229, 0.240) | 0.213 (0.208, 0.218) | 0.295 (0.289, 0.300) | 0.269 (0.263, 0.274) | 0.295 (0.290, 0.300) | 1.336% (-0.058%, 3.044%) | 0.067 |
|  | Inflammatory heart disease | Female | 0.236 (0.216, 0.256) | 0.139 (0.132, 0.147) | 0.139 (0.127, 0.150) | 0.151 (0.145, 0.158) | 0.152 (0.145, 0.158) | -0.954% (-3.417%, 2.162%) | 0.610 |
|  |  | Male | 0.159 (0.151, 0.166) | 0.212 (0.204, 0.221) | 0.172 (0.165, 0.179) | 0.339 (0.326, 0.351) | 0.277 (0.268, 0.286) | 2.965% (-0.008%, 6.415%) | 0.051 |
|  |  | Person | 0.177 (0.169, 0.184) | 0.175 (0.169, 0.180) | 0.139 (0.134, 0.144) | 0.234 (0.228, 0.241) | 0.213 (0.207, 0.219) | 1.427% (-1.230%, 4.986%) | 0.320 |
|  | Non-rheumatic valvular disease | Female | 0.361 (0.352, 0.371) | 0.409 (0.400, 0.418) | 0.354 (0.346, 0.362) | 0.317 (0.310, 0.325) | 0.319 (0.312, 0.325) | -0.960% (-3.089%, 1.412%) | 0.314 |
|  |  | Male | 0.567 (0.554, 0.580) | 0.645 (0.628, 0.661) | 0.547 (0.536, 0.558) | 0.441 (0.432, 0.450) | 0.419 (0.409, 0.428) | -1.764% (-4.252%, 1.026%) | 0.198 |
|  |  | Person | 0.459 (0.451, 0.467) | 0.523 (0.514, 0.533) | 0.446 (0.439, 0.452) | 0.376 (0.371, 0.382) | 0.367 (0.361, 0.373) | -1.398% (-3.729%, 1.312%) | 0.251 |
|  | Peripheral vascular disease | Female | 0.220 (0.213, 0.228) | 0.133 (0.128, 0.138) | 0.126 (0.121, 0.130) | 0.141 (0.136, 0.146) | 0.122 (0.118, 0.126) | -2.676% (-4.167%, -1.235%) | < 0.001 |
|  |  | Male | 0.383 (0.367, 0.399) | 0.228 (0.220, 0.235) | 0.209 (0.203, 0.216) | 0.229 (0.222, 0.236) | 0.197 (0.191, 0.203) | -2.521% (-4.996%, 0.425%) | 0.100 |
|  |  | Person | 0.298 (0.289, 0.306) | 0.177 (0.172, 0.181) | 0.165 (0.161, 0.169) | 0.182 (0.178, 0.187) | 0.157 (0.154, 0.161) | -2.483% (-4.737%, 0.145%) | 0.065 |
|  | Rheumatic heart disease (including acute rheumatic fever) | Female | 0.223 (0.215, 0.231) | 0.199 (0.192, 0.207) | 0.179 (0.173, 0.185) | 0.153 (0.147, 0.159) | 0.125 (0.120, 0.130) | -2.727% (-4.177%, -1.250%) | < 0.001 |
|  |  | Male | 0.143 (0.136, 0.150) | 0.116 (0.111, 0.122) | 0.133 (0.127, 0.139) | 0.119 (0.113, 0.124) | 0.091 (0.087, 0.096) | -1.783% (-4.324%, 0.975%) | 0.194 |
|  |  | Person | 0.185 (0.179, 0.190) | 0.159 (0.154, 0.163) | 0.157 (0.153, 0.161) | 0.137 (0.133, 0.141) | 0.109 (0.106, 0.112) | -2.356% (-3.696%, -0.965%) | < 0.001 |
|  | Stroke | Female | 3.951 (3.918, 3.985) | 2.822 (2.795, 2.849) | 2.259 (2.238, 2.280) | 2.223 (2.199, 2.247) | 1.742 (1.723, 1.760) | -3.869% (-4.504%, -3.290%) | < 0.001 |
|  |  | Male | 4.804 (4.766, 4.842) | 3.368 (3.339, 3.398) | 2.729 (2.705, 2.753) | 2.651 (2.628, 2.675) | 2.179 (2.158, 2.199) | -3.759% (-4.604%, -2.967%) | < 0.001 |
|  |  | Person | 4.343 (4.319, 4.368) | 3.091 (3.071, 3.110) | 2.489 (2.474, 2.505) | 2.428 (2.412, 2.445) | 1.952 (1.938, 1.965) | -3.795% (-4.408%, -3.234%) | < 0.001 |
| **Endocrine disorders** |  |  |  |  |  |  |  |  |  |
|  | Type 2 diabetes mellitus | Female | 0.966 (0.950, 0.982) | 0.860 (0.847, 0.873) | 0.624 (0.613, 0.635) | 0.707 (0.696, 0.719) | 0.616 (0.606, 0.626) | -2.228% (-3.442%, -0.952%) | < 0.001 |
|  |  | Male | 1.808 (1.784, 1.831) | 1.483 (1.464, 1.502) | 1.219 (1.202, 1.235) | 1.375 (1.358, 1.392) | 1.288 (1.273, 1.303) | -1.577% (-2.688%, -0.419%) | 0.002 |
|  |  | Person | 1.366 (1.352, 1.380) | 1.158 (1.146, 1.169) | 0.911 (0.902, 0.921) | 1.029 (1.019, 1.039) | 0.938 (0.930, 0.947) | -1.797% (-2.979%, -0.508%) | < 0.001 |
| **Kidney and urinary diseases** |  |  |  |  |  |  |  |  |  |
|  | Chronic kidney disease | Female | 0.641 (0.628, 0.654) | 0.953 (0.927, 0.978) | 1.241 (1.208, 1.273) | 0.796 (0.775, 0.818) | 0.849 (0.830, 0.869) | 1.435% (-1.223%, 3.924%) | 0.324 |
|  |  | Male | 1.052 (1.032, 1.072) | 1.116 (1.088, 1.144) | 1.403 (1.379, 1.426) | 1.031 (1.008, 1.053) | 1.167 (1.144, 1.189) | 0.459% (-1.276%, 2.089%) | 0.478 |
|  |  | Person | 0.830 (0.819, 0.842) | 1.018 (1.000, 1.037) | 1.307 (1.287, 1.327) | 0.906 (0.891, 0.922) | 1.000 (0.985, 1.015) | 0.882% (-1.178%, 2.821%) | 0.361 |

**Supplementary Table S2.** ASRs of YLL for non-communicable diseases in Australia by disease and sex from 2003 to 2024.

Abbreviations: ASR, age-standardized rate per 1,000 population. YLL, years of life lost. APC, annual percentage change. CI, confidence interval. UI, uncertainty interval.

**ASR**


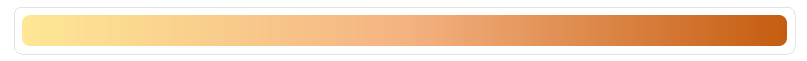
0.000 17.021

| Disease Group | Disease | Sex | 2003 ASR (95% UI) | 2011 ASR (95% UI) | 2015 ASR (95% UI) | 2018 ASR (95% UI) | 2024 ASR (95% UI) | 2003-2024 APC (95% CI） | P |
| --- | --- | --- | --- | --- | --- | --- | --- | --- | --- |
| **Cancer and other neoplasms** |  |  |  |  |  |  |  |  |  |
|  | Bowel cancer | Female | 0.146 (0.140, 0.152) | 0.138 (0.132, 0.143) | 0.129 (0.123, 0.134) | 0.124 (0.119, 0.129) | 0.114 (0.109, 0.118) | -1.221% (-1.661%, -0.747%) | < 0.001 |
|  |  | Male | 0.219 (0.211, 0.227) | 0.201 (0.194, 0.208) | 0.184 (0.178, 0.191) | 0.175 (0.169, 0.181) | 0.155 (0.15, 0.16) | -1.670% (-2.353%, -0.921%) | < 0.001 |
|  |  | Person | 0.179 (0.174, 0.184) | 0.167 (0.163, 0.172) | 0.154 (0.15, 0.158) | 0.148 (0.144, 0.152) | 0.133 (0.13, 0.136) | -1.437% (-2.102%, -0.708%) | < 0.001 |
|  | Breast cancer | Female | 0.495 (0.483, 0.507) | 0.502 (0.491, 0.513) | 0.505 (0.495, 0.516) | 0.492 (0.482, 0.503) | 0.491 (0.481, 0.501) | -0.068% (-0.289%, 0.167%) | 0.597 |
|  |  | Male | 0.004 (0.003, 0.005) | 0.005 (0.003, 0.006) | 0.005 (0.004, 0.006) | 0.005 (0.004, 0.006) | 0.005 (0.004, 0.006) | 1.092% (0.324%, 1.948%) | 0.004 |
|  |  | Person | 0.258 (0.252, 0.264) | 0.261 (0.255, 0.267) | 0.263 (0.258, 0.269) | 0.257 (0.252, 0.262) | 0.257 (0.252, 0.262) | -0.056% (-0.226%, 0.130%) | 0.574 |
|  | Lung cancer | Female | 0.053 (0.049, 0.057) | 0.06 (0.056, 0.063) | 0.064 (0.06, 0.067) | 0.064 (0.06, 0.067) | 0.068 (0.064, 0.071) | 1.109% (0.751%, 1.541%) | < 0.001 |
|  |  | Male | 0.109 (0.104, 0.115) | 0.099 (0.094, 0.104) | 0.092 (0.088, 0.097) | 0.089 (0.085, 0.093) | 0.081 (0.078, 0.085) | -1.423% (-1.516%, -1.328%) | < 0.001 |
|  |  | Person | 0.078 (0.074, 0.081) | 0.077 (0.074, 0.08) | 0.076 (0.074, 0.079) | 0.075 (0.072, 0.078) | 0.073 (0.071, 0.076) | -0.273% (-0.475%, -0.042%) | 0.012 |
|  | Oesophageal cancer | Female | 0.007 (0.006, 0.009) | 0.006 (0.005, 0.007) | 0.006 (0.005, 0.007) | 0.005 (0.004, 0.006) | 0.005 (0.004, 0.006) | -1.932% (-2.386%, -1.446%) | < 0.001 |
|  |  | Male | 0.020 (0.018, 0.022) | 0.02 (0.018, 0.022) | 0.019 (0.017, 0.021) | 0.019 (0.017, 0.021) | 0.018 (0.016, 0.02) | -0.638% (-0.957%, -0.323%) | < 0.001 |
|  |  | Person | 0.013 (0.012, 0.014) | 0.013 (0.012, 0.014) | 0.012 (0.011, 0.013) | 0.012 (0.011, 0.013) | 0.011 (0.01, 0.012) | -0.910% (-1.156%, -0.660%) | < 0.001 |
| **Cardiovascular diseases** |  |  |  |  |  |  |  |  |  |
|  | Aortic aneurysm | Female | 0 (0, 0) | 0 (0, 0) | 0 (0, 0) | 0 (0, 0) | 0 (0, 0) | 0 | - |
|  |  | Male | 0.001 (0.001, 0.002) | 0.001 (0, 0.001) | 0 (0, 0.001) | 0 (0, 0.001) | 0 (0, 0) | -8.302% (-9.149%, -7.648%) | < 0.001 |
|  |  | Person | 0.001 (0, 0.001) | 0 (0, 0) | 0 (0, 0) | 0 (0, 0) | 0 (0, 0) | -8.416% (-9.294%, -7.720%) | < 0.001 |
|  | Atrial fibrillation and flutter | Female | 0.542 (0.530, 0.553) | 0.554 (0.543, 0.565) | 0.634 (0.623, 0.644) | 0.689 (0.678, 0.700) | 0.677 (0.667, 0.686) | 1.248% (-0.065%, 2.917%) | 0.071 |
|  |  | Male | 0.919 (0.903, 0.936) | 0.945 (0.930, 0.961) | 1.075 (1.059, 1.090) | 1.175 (1.160, 1.190) | 1.164 (1.150, 1.178) | 1.319% (0.038%, 2.945%) | 0.041 |
|  |  | Person | 0.724 (0.714, 0.734) | 0.745 (0.735, 0.754) | 0.848 (0.839, 0.857) | 0.924 (0.915, 0.933) | 0.912 (0.903, 0.921) | 1.291% (0.001%, 2.920%) | 0.049 |
|  | Cardiomyopathy | Female | 0.057 (0.054, 0.061) | 0.051 (0.048, 0.054) | 0.049 (0.046, 0.052) | 0.050 (0.047, 0.053) | 0.048 (0.046, 0.051) | -0.746% (-1.158%, -0.305%) | < 0.001 |
|  |  | Male | 0.118 (0.112, 0.124) | 0.100 (0.095, 0.105) | 0.095 (0.091, 0.100) | 0.098 (0.093, 0.102) | 0.095 (0.091, 0.099) | -0.946% (-2.114%, 0.387%) | 0.161 |
|  |  | Person | 0.083 (0.080, 0.086) | 0.072 (0.070, 0.075) | 0.070 (0.067, 0.073) | 0.072 (0.07, 0.075) | 0.070 (0.067, 0.072) | -0.748% (-1.486%, 0.062%) | 0.076 |
|  | Coronary heart disease | Female | 1.705 (1.684, 1.725) | 1.299 (1.283, 1.315) | 1.065 (1.051, 1.079) | 0.998 (0.985, 1.011) | 0.802 (0.791, 0.812) | -3.561% (-3.847%, -3.281%) | < 0.001 |
|  |  | Male | 3.382 (3.350, 3.414) | 2.617 (2.592, 2.642) | 2.183 (2.162, 2.205) | 2.061 (2.041, 2.081) | 1.690 (1.673, 1.707) | -3.276% (-3.564%, -2.975%) | < 0.001 |
|  |  | Person | 2.483 (2.465, 2.501) | 1.922 (1.908, 1.937) | 1.596 (1.583, 1.608) | 1.503 (1.491, 1.515) | 1.224 (1.214, 1.234) | -3.341% (-3.616%, -3.067%) | < 0.001 |
|  | Hypertensive heart disease | Female | 0.004 (0.003, 0.005) | 0.004 (0.003, 0.004) | 0.003 (0.003, 0.004) | 0.003 (0.003, 0.004) | 0.003 (0.003, 0.004) | -1.028% (-1.575%, -0.432%) | < 0.001 |
|  |  | Male | 0.003 (0.003, 0.004) | 0.003 (0.002, 0.004) | 0.003 (0.002, 0.003) | 0.003 (0.002, 0.003) | 0.003 (0.002, 0.003) | -1.294% (-2.580%, 0.136%) | 0.076 |
|  |  | Person | 0.004 (0.003, 0.005) | 0.003 (0.003, 0.004) | 0.003 (0.003, 0.004) | 0.003 (0.003, 0.004) | 0.003 (0.003, 0.003) | -1.249% (-2.093%, -0.299%) | 0.003 |
|  | Inflammatory heart disease | Female | 0.029 (0.026, 0.032) | 0.026 (0.024, 0.028) | 0.025 (0.023, 0.027) | 0.026 (0.024, 0.028) | 0.025 (0.023, 0.027) | -0.699% (-1.048%, -0.333%) | < 0.001 |
|  |  | Male | 0.044 (0.040, 0.047) | 0.037 (0.034, 0.040) | 0.036 (0.033, 0.038) | 0.036 (0.034, 0.039) | 0.035 (0.033, 0.038) | -0.942% (-1.969%, 0.212%) | 0.110 |
|  |  | Person | 0.035 (0.033, 0.037) | 0.031 (0.029, 0.033) | 0.030 (0.028, 0.031) | 0.031 (0.029, 0.032) | 0.030 (0.028, 0.031) | -0.707% (-1.340%, -0.018%) | 0.042 |
|  | Non-rheumatic valvular disease | Female | 0.305 (0.296, 0.314) | 0.225 (0.218, 0.232) | 0.206 (0.199, 0.212) | 0.202 (0.196, 0.208) | 0.184 (0.179, 0.19) | -2.333% (-4.098%, -0.421%) | 0.007 |
|  |  | Male | 0.357 (0.347, 0.367) | 0.294 (0.286, 0.302) | 0.281 (0.274, 0.289) | 0.283 (0.275, 0.29) | 0.269 (0.262, 0.276) | -1.256% (-2.570%, 0.256%) | 0.108 |
|  |  | Person | 0.326 (0.319, 0.332) | 0.256 (0.251, 0.261) | 0.241 (0.236, 0.246) | 0.24 (0.235, 0.244) | 0.224 (0.220, 0.228) | -1.707% (-3.234%, 0.001%) | 0.050 |
|  | Peripheral vascular disease | Female | 0.048 (0.044, 0.051) | 0.041 (0.038, 0.044) | 0.031 (0.028, 0.033) | 0.028 (0.026, 0.031) | 0.023 (0.021, 0.025) | -3.559% (-4.756%, -2.432%) | < 0.001 |
|  |  | Male | 0.084 (0.079, 0.089) | 0.075 (0.071, 0.080) | 0.054 (0.050, 0.057) | 0.047 (0.044, 0.051) | 0.038 (0.035, 0.040) | -3.952% (-5.129%, -2.868%) | < 0.001 |
|  |  | Person | 0.064 (0.061, 0.067) | 0.057 (0.054, 0.06) | 0.042 (0.04, 0.044) | 0.038 (0.036, 0.040) | 0.03 (0.028, 0.0320) | -3.752% (-4.767%, -2.821%) | < 0.001 |
|  | Rheumatic heart disease (including acute rheumatic fever) | Female | 0.040 (0.037, 0.044) | 0.033 (0.03, 0.036) | 0.027 (0.025, 0.03) | 0.028 (0.025, 0.031) | 0.024 (0.022, 0.026) | -2.472% (-3.066%, -1.857%) | < 0.001 |
|  |  | Male | 0.022 (0.019, 0.025) | 0.019 (0.017, 0.021) | 0.016 (0.014, 0.018) | 0.018 (0.015, 0.020) | 0.015 (0.013, 0.017) | -1.724% (-2.402%, -1.021%) | < 0.001 |
|  |  | Person | 0.032 (0.029, 0.034) | 0.026 (0.024, 0.028) | 0.022 (0.02, 0.024) | 0.023 (0.021, 0.025) | 0.020 (0.018, 0.021) | -2.207% (-2.808%, -1.584%) | < 0.001 |
|  | Stroke | Female | 0.372 (0.362, 0.381) | 0.327 (0.318, 0.335) | 0.306 (0.298, 0.314) | 0.315 (0.307, 0.323) | 0.286 (0.279, 0.293) | -1.184% (-1.832%, -0.488%) | < 0.001 |
|  |  | Male | 0.557 (0.544, 0.570) | 0.476 (0.465, 0.486) | 0.450 (0.44, 0.46) | 0.461 (0.451, 0.470) | 0.420 (0.411, 0.428) | -1.256% (-1.770%, -0.703%) | < 0.001 |
|  |  | Person | 0.455 (0.447, 0.463) | 0.396 (0.389, 0.402) | 0.374 (0.368, 0.38) | 0.384 (0.378, 0.390) | 0.349 (0.344, 0.355) | -1.180% (-1.965%, -0.317%) | 0.002 |
| **Endocrine disorders** |  |  |  |  |  |  |  |  |  |
|  | Type 2 diabetes mellitus | Female | 1.237 (1.218, 1.257) | 1.574 (1.555, 1.594) | 1.730 (1.710, 1.75) | 1.480 (1.462, 1.498) | 1.465 (1.448, 1.481) | 0.436% (-2.589%, 4.012%) | 0.582 |
|  |  | Male | 1.700 (1.677, 1.723) | 1.975 (1.953, 1.998) | 2.214 (2.191, 2.237) | 2.061 (2.040, 2.083) | 2.085 (2.065, 2.105) | 0.841% (-0.703%, 2.691%) | 0.239 |
|  |  | Person | 1.458 (1.443, 1.473) | 1.766 (1.751, 1.781) | 1.958 (1.943, 1.974) | 1.764 (1.75, 1.778) | 1.764 (1.751, 1.777) | 0.676% (-1.077%, 2.762%) | 0.335 |
| **Kidney and urinary diseases** |  |  |  |  |  |  |  |  |  |
|  | Chronic kidney disease | Female | 0.289 (0.280, 0.297) | 0.314 (0.305, 0.322) | 0.316 (0.308, 0.324) | 0.319 (0.311, 0.327) | 0.330 (0.323, 0.338) | 0.590% (0.307%, 0.917%) | < 0.001 |
|  |  | Male | 0.330 (0.320, 0.340) | 0.362 (0.353, 0.372) | 0.371 (0.361, 0.380) | 0.372 (0.363, 0.381) | 0.388 (0.380, 0.397) | 0.715% (0.365%, 1.120%) | < 0.001 |
|  |  | Person | 0.310 (0.303, 0.316) | 0.338 (0.331, 0.344) | 0.343 (0.337, 0.349) | 0.345 (0.340, 0.351) | 0.359 (0.353, 0.365) | 0.653% (0.298%, 1.064%) | < 0.001 |

**Supplementary Table S3.** ASRs of YLD for non-communicable diseases in Australia by disease and sex from 2003 to 2024.

Abbreviations: ASR, age-standardized rate per 1,000 population. YLD, years lived with disability. APC, annual percentage change. CI, confidence interval. UI, uncertainty interval.

**ASR**


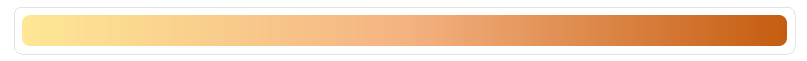
0.000 3.382
